# Revisiting the Role of Physical Confinement and Chemical Regulation of 3D Hosts for Dendrite-Free Li Metal Anode

**DOI:** 10.1007/s40820-022-00932-3

**Published:** 2022-09-14

**Authors:** Shufen Ye, Xingjia Chen, Rui Zhang, Yu Jiang, Fanyang Huang, Huijuan Huang, Yu Yao, Shuhong Jiao, Xiang Chen, Qiang Zhang, Yan Yu

**Affiliations:** 1 Hefei National Center for Physical Sciences at the Microscale, Department of Materials Science and Engineering, iChEM (Collaborative Innovation Center of Chemistry for Energy Materials), CAS Key Laboratory of Materials for Energy Conversion, University of Science and Technology of China, Hefei, 230026 Anhui People’s Republic of China; 2 Advanced Research Institute of Multidisciplinary Science, Beijing Institute of Technology, Beijing, 100081 People’s Republic of China; 3 School of Materials Science and Engineering, Anhui University, Hefei, 230601 Anhui People’s Republic of China; 4 Beijing Key Laboratory of Green Chemical Reaction Engineering and Technology, Department of Chemical Engineering, Tsinghua University, Beijing, 100084 People’s Republic of China; 5 National Synchrotron Radiation Laboratory, Hefei, 230026 Anhui People’s Republic of China

**Keywords:** Li metal anodes, 3D carbon framework, Ni-based nanosheets, Physical morphology confinement, Chemical adsorption/diffusion regulation

## Abstract

Lithium metal anode has been demonstrated as the most promising anode for lithium batteries because of its high theoretical capacity, but infinite volume change and dendritic growth during Li electrodeposition have prevented its practical applications. Both physical morphology confinement and chemical adsorption/diffusion regulation are two crucial approaches to designing lithiophilic materials to alleviate dendrite of Li metal anode. However, their roles in suppressing dendrite growth for long-life Li anode are not fully understood yet. Herein, three different Ni-based nanosheet arrays (NiO-NS, Ni_3_N-NS, and Ni_5_P_4_-NS) on carbon cloth as proof-of-concept lithiophilic frameworks are proposed for Li metal anodes. The two-dimensional nanoarray is more promising to facilitate uniform Li^+^ flow and electric field. Compared with the NiO-NS and the Ni_5_P_4_-NS, the Ni_3_N-NS on carbon cloth after reacting with molten Li (Li-Ni/Li_3_N-NS@CC) can afford the strongest adsorption to Li^+^ and the most rapid Li^+^ diffusion path. Therefore, the Li-Ni/Li_3_N-NS@CC electrode realizes the lowest overpotential and the most excellent electrochemical performance (60 mA cm^−2^ and 60 mAh cm^−2^ for 1000 h). Furthermore, a remarkable full battery (LiFePO_4_||Li-Ni/Li_3_N-NS@CC) reaches 300 cycles at 2C. This research provides valuable insight into designing dendrite-free alkali metal batteries.

**Supplementary Information:**

The online version contains supplementary material available at 10.1007/s40820-022-00932-3.

## Highlights

- The two-dimensional nanoarray with excellent physical confinement is more promising to facilitate uniform Li^+^ flow and electric field.
- Li_3_N with superior chemical regulation provides nucleation sites, accelerates the replenishment of consumed Li^+^, and achieves dendrite-free morphology.
- The synergistic effect of physical confinement and chemical regulation achieves superior electrochemical performance at high current density and areal capacity.

**Supplementary Information:**

The online version contains supplementary material available at 10.1007/s40820-022-00932-3.

## Graphical abstract

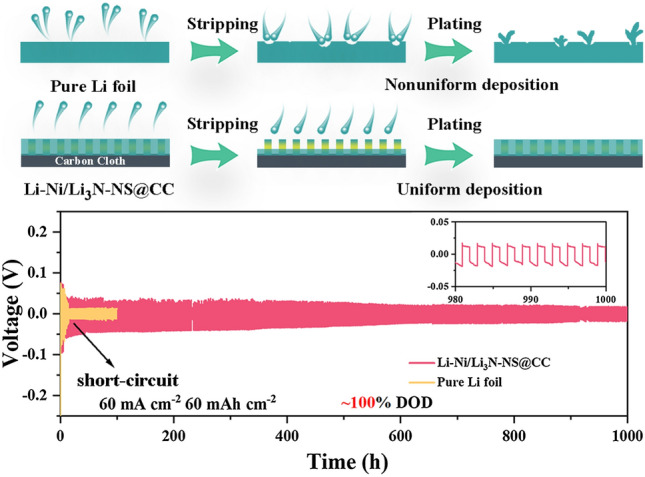

## Introduction

Given the lightweight (0.534 g cm^−3^), high theoretical capacity (3860 mAh g^−1^), and ultralow redox potential (− 3.040 V versus standard hydrogen electrode) of Li metal, Li metal batteries (e.g., Li–S and Li–CO_2_ batteries) with low cost can realize extremely high theoretical energy density for satisfying the targets of future electric vehicles [1]. However, the application of lithium metal anode (LMA) is still grievously obstructed by unmanageable dendrite growth and infinite volume fluctuation, giving rise to the unavoidable side reaction, low Coulombic efficiency (CE), and even safety risks [2–6].

Some progress has been made to alleviate infinite volume expansion and form the dendrite-free LMA during cycling, such as adjusting the electrolyte formulations [7–15], constructing an artificial interface layer on LMA [16–19], and designing solid-state electrolytes [20–24]. Although these strategies can prevent the Li dendrite growth to some degree, the dramatical electrode volume change generated by “hostless” Li plating/stripping still prevails and will destroy the solid electrolyte interface (SEI), which ineluctably facilitates dendrite growth and exacerbates the depletion of Li metal, especially at high current densities and cycling capacities [25–27]. The three-dimensional (3D) framework can reform the traditional nucleation and growth mode at the source, which not only can accommodate the large volume change but also can control the nucleation of Li^+^ to obtain stable Li deposition and growth [28–31]. The 3D carbon frameworks with lightweight, flexibility, and abundant voids are the better option compared with widely investigated 3D metal foams. However, considering the inferior lithiophilicity of carbon-based skeletons, they are unattractive for manipulating Li nucleation and continuous Li growth to achieve high areal capacity and the long-cycle performance of LMA.

Inspired by selective Li nucleation/growth through heterogeneous seeds presented by Cui’s group [32], various lithiophilic materials (such as oxides [33–35], nitrides [36, 37], and phosphides [38, 39]) have been embedded into carbon frameworks to reduce the overpotential of Li nucleation and regulate uniform Li nucleation/growth. Besides, some lithiophilic compounds can react with molten Li to produce Li-based compounds (like, Li_3_N, Li_2_O, and Li_3_P) and metal, which facilitates the Li-ion transfer kinetics (e.g., Li^+^ conductivity ≈ 10^−3^ S cm^−1^ for Li_3_N), and electron conduction [40–42]. Meanwhile, different morphologies of lithiophilic materials have been designed on the carbon matrix, like, zero-dimensional (0D) nanoparticles [43–46], one-dimensional (1D) nanowires [47–49], two-dimensional (2D) nanosheets [33, 50, 51], etc. For example, Manthiram et al. [52] prepared the 0D Mo_2_N nanoparticles-modified carbon nanofiber (CNF) framework as a Li host, which operated over 1500 h at 6 mA cm^−2^/6 mAh cm^−2^. Wen et al. combined 1D Cu_3_P nanowires with Cu foil as a 3D Li host, working stably over 450 h at 2 mA cm^−2^/2 mAh cm^−2^ [38]. Yang et al. [33] reported that 2D MnZnO nanosheets/CNF infused with molten Li maintained 40 h at 50 mA cm^−2^ and 10 mAh cm^−2^. Nevertheless, the modified Li anodes with high areal capacities (> 10 mAh cm^−2^) can only be performed (< 1000 h) at limited current densities (< 10 mA cm^−2^) [53–55]. Therefore, a rational design of lithiophilic material remains unclear for protecting Li metal anode toward practical application (especially at ultrahigh current density and areal capacity).

In this contribution, we synthesize three different Ni-based nanosheets (NiO-NS, Ni_3_N-NS, and Ni_5_P_4_-NS) on carbon cloth as the proof-of-concept lithiophilic framework to demonstrate the effect of physical morphology confinement and chemical adsorption/diffusion regulation. Finite element method (FEM) simulation confirms that compared with 0D nanoparticles and 1D nanowires, the carbon framework covered with 2D nanosheets provides promising physical confinement, homogenizing Li^+^ flow, and lowering activation overpotential. Density functional theory (DFT) calculations reveal that Li_3_N shows the lowest ion diffusion energy barrier and the strongest adsorption energy toward Li^+^ in comparison with Li_2_O and Li_3_P. Li_3_N with superior chemical regulation provides nucleation sites, accelerates the replenishment of consumed lithium ions, and achieves dendrite-free morphology. Benefiting from the guidance of theoretical simulations, the Ni_3_N nanosheet (Ni_3_N-NS) on carbon cloth after infiltrating with molten Li (denoted as Li-Ni/Li_3_N-NS@CC) delivers low overpotential (~ 34 mV) and outstanding long-cycle performance (a lifespan of 1000 h at a current density of 60 mA cm^−2^ and a capacity of 60 mAh cm^−2^) in symmetrical batteries. Moreover, the full battery (Li-Ni/Li_3_N-NS@CC||LiFePO_4_) shows an average CE of 99.8% and remarkable capacity retention (93.9% after 300 cycles at 2C) with a mass loading as high as 9.0 mg cm^−2^.

## Experiment Section

### Synthesis of Ni-Precursor-NS@CC

Firstly, carbon cloth was annealed at 600 °C for 1 h in a muffle furnace. Secondly, 0.03 mol NiCl_2_·6H_2_O, 0.15 mol urea, and 0.06 mol NH_4_F were dissolved in 30 mL of deionized water and thoroughly mixed under magnetic stirring. The solution was then transferred into a 50 mL Teflon-lined stainless-steel autoclave and as-prepared carbon cloth was put into the solution. Then the autoclave was heated at 120 °C for 4 h. After heating, the sample was washed with anhydrous ethanol and deionized water. Finally, the sample was treated by vacuum drying at 60 °C to obtain Ni-precursor-NS@CC.

### Synthesis of Ni-Based Nanosheet Decorated on CC

The Ni-precursor-NS@CC was further heated in a muffle furnace at 400 °C for 1 h to obtain NiO-NS@CC. The Ni-precursor-NS@CC was annealed at 350 °C for 2 h in NH_3_ gas to obtain Ni_3_N-NS@CC. The Ni-precursor-NS@CC and excess sodium hypophosphite (NaH_2_PO_2_·H_2_O) were separately placed in downstream and upstream of the tube furnace, respectively, and heated at 350 °C for 2 h under Ar atmosphere to obtain Ni_5_P_4_-NS@CC. The weight ratio of NaH_2_PO_2_·H_2_O and Ni-precursor-NS@CC was fixed at 10:1.

### Fabrication of Composite Li Metal Anodes

A facile molten infusion method at 400 °C was applied to immerse molten Li into NiO-NS@CC, Ni_3_N-NS@CC, and Ni_5_P_4_-NS@CC to obtain Li-Ni/Li_2_O-NS@CC, Li-Ni/Li_3_N-NS@CC, Li-Ni/Li_3_P-NS@CC, respectively. Li@CC electrode was obtained by immersing the carbon cloth after annealing at 600 °C into molten lithium. The whole process was operated in an Ar-filled glove box (H_2_O < 0.1 ppm; O_2_ < 0.1 ppm).

### Materials Characterization

The morphologies were characterized using tungsten filament scanning electron microscopy (SEM, JEOL, JSM-6360LA, CIQTEK-SEM3100). Phase structures of all samples were characterized by employing x-ray diffraction (XRD, Rigaku, TTR-III) with a Cu Kα radiation (*λ* = 0.15418 nm). The easily oxidized samples for XRD measurements were sealed in the Kapton film to avoid oxidation. The atomic force microscopy (AFM) images of metal anodes were detected by AFM in the glovebox (Bruker).

### Electrochemical Measurements

CR2032-type coin cells were assembled in an Ar-filled glove box (H_2_O < 0.1 ppm; O_2_ < 0.1 ppm). Li symmetrical batteries were assembled with two Li foils or two composite Li metal electrodes with a diameter of 10 mm. In symmetrical batteries and Li-Cu batteries, 1.0 M lithium bistrifluoro-methanesulfonylimide (LiTFSI) in 1,3-Dioxolane (DOL) and Methoxymethane (DME) (volume ratio 1:1) with 1% lithium nitrate was employed as the electrolyte. For consistency, the amount of the electrolyte for all batteries was approximately 80 μL. The LiFePO_4_ (LFP) was employed as cathode while the fabricated composite Li metal electrode or pure Li foil was used as the anode in a full battery. The cathode diameter was 12 mm. The ratio of LFP, Super P and binder is 8:1:1. The electrolyte solution used in LFP full battery was 1.0 M LiPF_6_ in ethylene carbonate (EC)/diethyl carbonate (DEC) (volume ratio 1:1) with 5.0 wt% fluoroethylene carbonate (FEC) additive. The amount of electrolyte is fixed at 40 μL. All batteries use microporous polypropylene film (Celgard, 2400) as the separator.

### Electrochemical Measurements

To evaluate the CE in half cells, a certain capacity of Li was deposited on the working electrode, and then Li was stripped until the voltage rose to 1 V. The LFP full batteries were charge/discharge cycled between 2.4 and 4.2 V. Tafel plots and electrochemical impedance spectroscopy (EIS) test were performed through an electrochemical workstation (CHI 660D, Chenhua Instrument Company, Shanghai, China). The frequency range of EIS was 10 mHz–100 kHz, while the perturbation amplitude was 5 mV (versus the open circuit potential). The electrochemical performances of symmetrical cells and full batteries were measured using Neware testing instrument (BTS-610).

### Computational Methods

All first-principles calculations were carried out based on the spin-polarized DFT framework by utilizing the Vienna Ab initio Simulation Package (VASP) 5.4.4 package [56, 57]. The projector-augmented wave pseudopotential [58, 59] was utilized to treat the core electrons, while the Perdew–Burke–Ernzerhof (PBE) exchange–correlation functional of the generalized gradient approximation (GGA) [60] was used for describing the electron interactions. The electronic wave functions were expanded on a plane-wave basis set with a kinetic energy cutoff of 500 eV. The convergence criterion for the electronic self-consistent cycle is fixed at 1 × 10^−5^ eV. The Brillouin zone is sampled with a 0.03 × 2π Å^−1^ spaced Γ-centered k-point grid with the Monkhorst–Pack scheme [61]. The structures of crystal Li_2_O, Li_3_N, and Li_3_P adopt the symmetry group of Fm-3 m (No. 225), P6/mmm (No. 191), and P63/mmc (No. 194), respectively, according to the experimental characterization results, which were further optimized with the force convergence criteria of 0.01 eV Å^−1^. To determine the optimal adsorption configurations of these secondary products toward Li^+^, slab models of 3 × 3 supercell for Li_2_O (111), 3 × 3 supercell for Li_3_N (001), 1 × 3 supercell for Li_3_P (101) containing 81, 72, 72 atoms were built, respectively. For the nickel model, a four-layer 4 × 4 × 1 supercell including 64 atoms in total was considered. Adsorption of lithium on 5 × 5 graphene was calculated for comparison. A vacuum space of 15 Å along the z-direction was included to avoid interactions between the periodic images. The atomic positions of the bottom half layers in slab models were fixed to their optimal bulk positions and the remaining atoms were fully relaxed until the maximum force on each atom was less than 0.03 eV Å^−1^ during optimization. The DFT-D3 method of Grimme was applied to better describe the van der Waals interactions [62]. The adsorption energies ($E_{{{\text{ads}}}}$) of Li on different surfaces were calculated by the following equation:

1 $$E_{{{\text{ads}}}} = E_{{{\text{total}}}} - E_{{{\text{surf}}}} - E_{{{\text{Li}}}}$$

where $E_{{{\text{total}}}}$, $E_{{{\text{surf}}}}$ and $E_{{{\text{Li}}}}$ are energies of the Li/surface, the pristine surface, and a Li atom in the lithium crystal, respectively.

The differential charge density distribution was drawn with an isosurface value of 0.002 e^−^/bohr^3^. The yellow surface corresponds to charge accumulation and the blue one corresponds to charge depletion. Li-ion migration energy barrier calculations were performed with the climbing image nudged-elastic band (CI-NEB) method [63].

### Finite Element Simulation

FEM was employed to investigate the distribution of Li-ion concentration and electric potential in with 2D nanosheets 1D nanowires, or 0D nanoparticles electrodes based on COMSOL Multiphysics 5.5 platform with the physical module “Tertiary Current Distribution, Nernst-Planck”.

A three-dimensional domain with a size of 4.0 × 8.0 × 8.0 μm^3^ is introduced. The surface of the current collector of the 2D nanosheets, 1D nanoarray nanowires and 0D nanoparticles model consists of sheet-like, wire-like and ball-like protrusions, respectively. 2D- and 1D-based types of protrusions have the same protrusion height (3.0 μm) and distribution density. A constant current density of 5.0 mA cm^−2^ is applied in these galvanostatic models. The diffusion coefficient of Li ions is set as 1.5 × 10^−10^ m^2^ s^−1^. The initial concentration of Li ions is set as 1 M.

## Results and Discussion

### Physical Morphology Confinement Analysis

**Fig. 1:**
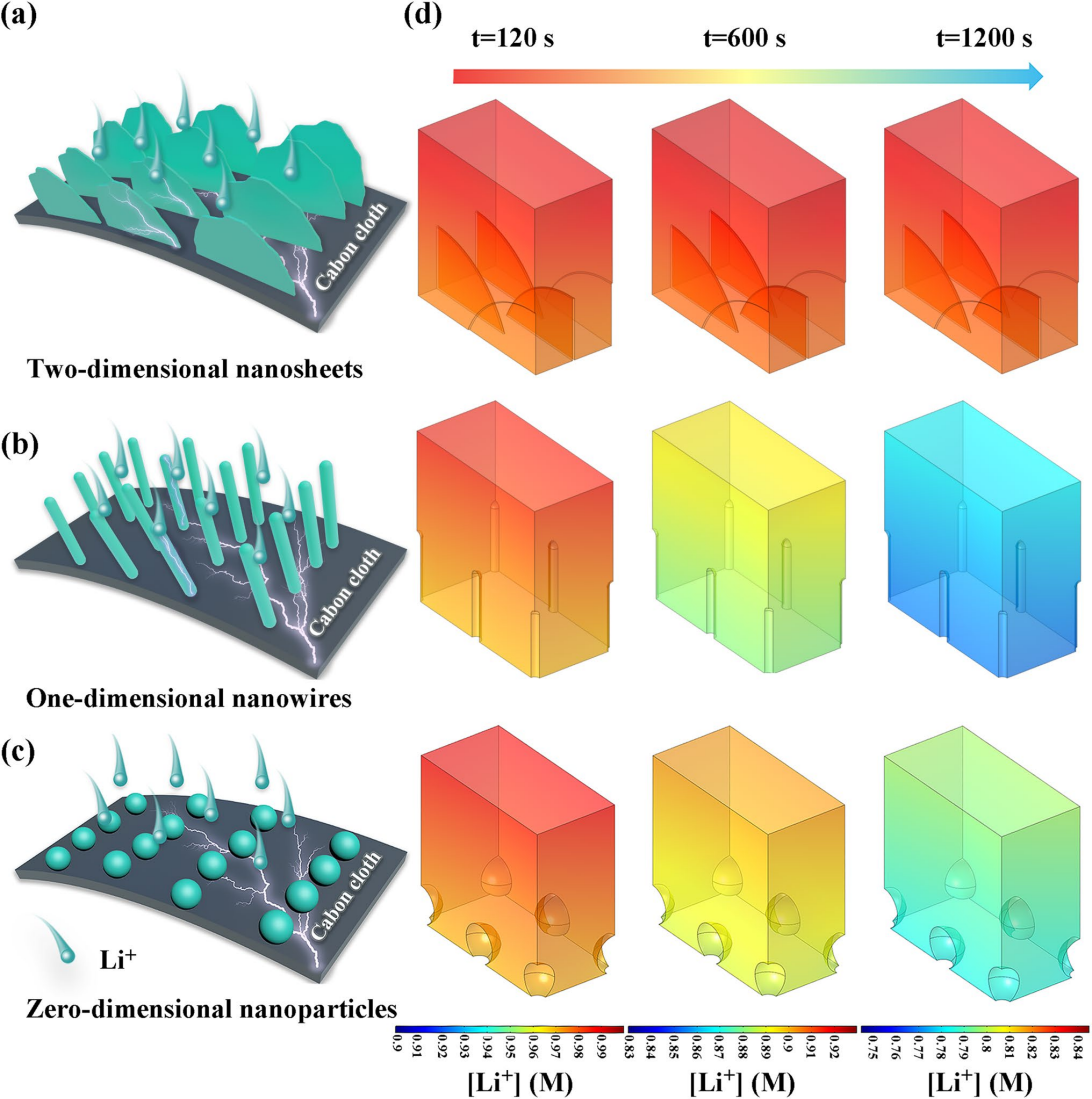
Schematic illustrations of Li^+^ plated on the carbon cloth decorated with **a** 2D nanosheets, **b** 1D nanowires, and **c** 0D nanoparticles. **d** The simulated results of Li^+^ concentration distribution in the electrolyte near 2D nanosheets, 1D nanowires, and 0D nanoparticles after different Li plating times

In order to reveal the effect of morphologies on the physical confinement of lithium deposition, FEM simulation is carried out to compare the Li^+^ concentration, electrolyte electric potential, and current line distributions near carbon cloth covered with 2D nanosheets (Fig. 1a), 1D nanowires (Fig. 1b), and 0D nanoparticles (Fig. 1c). In case of the same deposition time, Li^+^ will be deposited more uniformly on the carbon skeleton with 2D nanosheets than that of the carbon skeleton with 1D nanowires and 0D nanoparticles, as shown in Fig. 1d. Especially, even after 1200 s of deposition, the concentration of Li^+^ nearly remains the same near the surface of the carbon skeleton coated with the 2D nanosheets, realizing dendrite-free deposition. Conversely, the concentration of Li^+^ shows a significant change around the carbon skeleton coated with the 1D nanowires and 0D nanoparticles, driving the growth of dendritic structures. Corresponding electrolyte electric potential and current line distribution (Fig. S1) give a further intuitive explanation. Considering that the electrode potential and the equilibrium potential for Li plating (vs. Li^+^/Li) are both 0 V, the electrolyte electric potential near the nanoarray/nanoparticle surface can be considered as the activation overpotential for Li plating in this model [64–66]. Carbon skeleton with 2D nanosheets also shows the smallest activation overpotential and the most uniform current line distribution than those of carbon skeleton with 1D nanowires and 0D nanoparticles, realizing uniform local current density and dendrite-free morphology for the nanosheet-based framework. Overall, FEM simulations show that the design of the 2D nanosheets is the most promising approach to inhibiting dendrite growth and achieving uniform deposition. With this idea in mind, carbon cloth decorated with Ni-based nanosheets after reacting with molten Li (Li-Ni/Li_m_X-NS@CC (X = O, N and P)) is developed herein.

### Preparation and Characterizations of Li-Ni/Li_m_X-NS@CC Electrodes

**Fig. 2:**
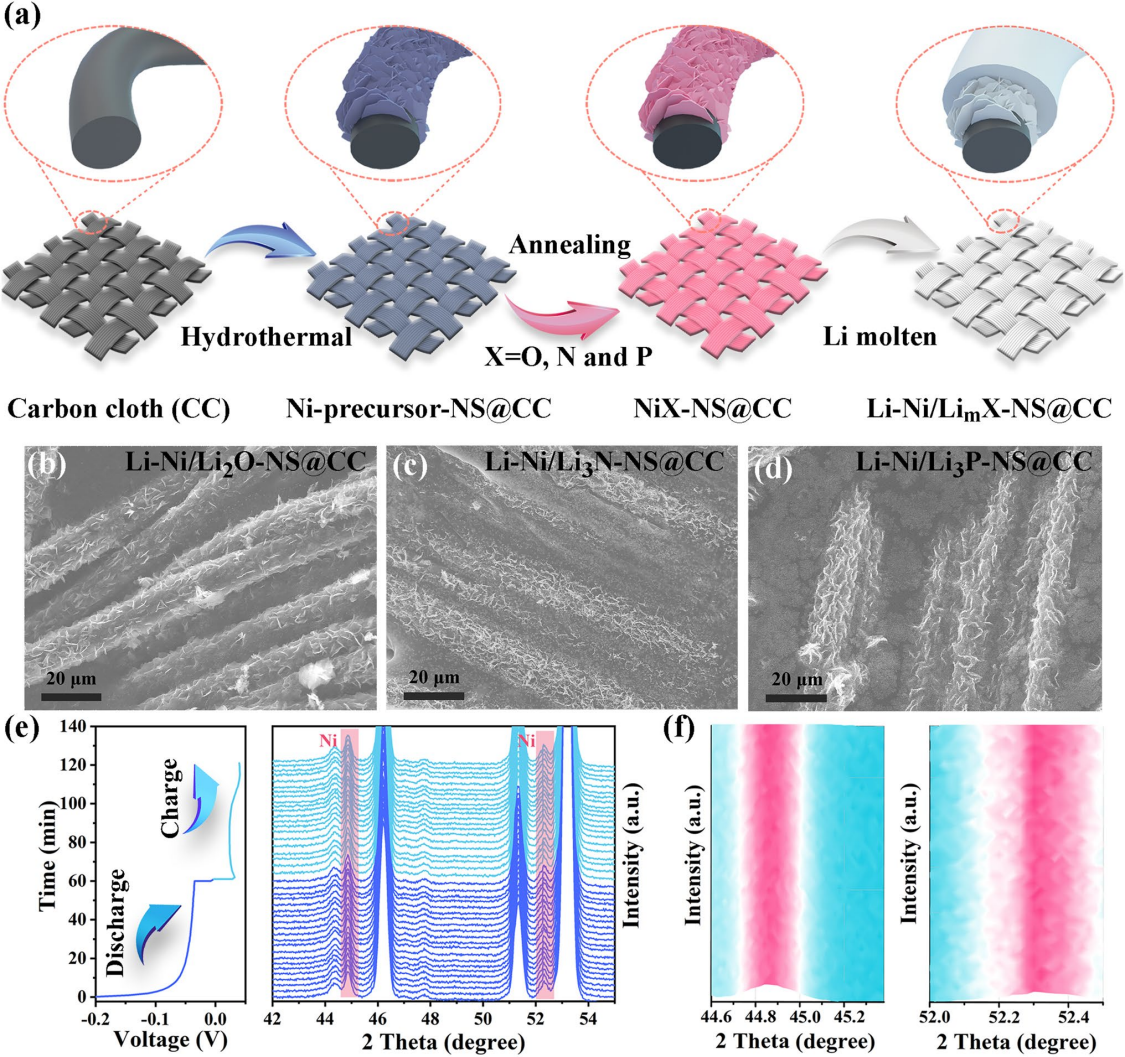
**a** Schematic diagram of the fabrication procedure of the Li-Ni/Li_m_X-NS@CC. The SEM images of **b** the Li-Ni/Li_2_O-NS@CC, **c** the Li-Ni/Li_3_N-NS@CC, and **d** the Li-Ni/Li_3_P-NS@CC. **e** In situ XRD patterns of the Li-Ni/Li_3_N-NS@CC electrode during the discharge and charge process. **f** Mountain-like peak images of Ni

The fabrication process of the Li-Ni/Li_m_X-NS@CC anode is schematically illustrated in Fig. 2a, mainly containing the growth of nanosheets and molten Li infusion. SEM images (Fig. S2) show that 2D Ni-precursor nanosheet (Ni-precursor-NS@CC) arrays are grown vertically on carbon cloth via a hydrothermal method and the thickness of nanosheets (Fig. S3) is about 30 nm. After calcination in air, as exhibited in Fig. S4, three main peaks at 37.24°, 43.24°, and 62.58° in XRD pattern are related to the (111), (200), and (220) planes of crystalline NiO, respectively, and uniform NiO nanosheets are distributed on carbon cloth. When the annealing atmosphere is NH_3_, four crystallization peaks in the XRD pattern (Fig. S5a) can be assigned to a hexagonal phase with the *P6*_3_22 space group (PDF#10-0280), indicating that a pure Ni_3_N phase is obtained. The Ni_3_N inherits the nanosheet morphology of the Ni-precursor. However, the nanosheets after sintering display porous microstructures (Fig. S5b) owing to the atomic diffusion and grain growth at 350 °C [67]. Similarly, after the phosphidation process, the Ni_5_P_4_ nanosheets (Fig. S6a) also show porous microstructures. All the diffraction peaks (Fig. S6b) except for the peak of carbon cloth at 25.8° are correlated to the standard hexagonal Ni_5_P_4_ (PDF#18-0883) without any detectable crystalline impurity. The thermogravimetric analysis (TGA) was performed to evaluate the Ni content (6.49%) in the Ni-precursor-NS@CC under air atmosphere (Fig. S7). And the loading of NiO, Ni_3_N and Ni_5_P_4_ is 8.25%, 7.09%, and 8.93%, respectively. And Table S1 shows the density and porosity of each electrode. Figure 2b–d shows that the nanosheet morphology of three Ni-based compounds remains after reacting with molten Li. Molten Li fills the interstice between each nanosheet and the void of the carbon cloth framework (Fig. S8). After immersing these compounds in molten Li, conversion reactions take place. Especially, NiO is reduced into Ni and Li_2_O (Fig. S9). Similarly, Ni_3_N is transferred into Ni and Li_3_N (Fig. S10), and the presence of Li_3_N is further confirmed by the Raman spectrum with a characteristic peak at ca. 580 cm^−1^ (Fig. S11) [68]. The specific capacity of molten Li in the Li-Ni/Li_3_N-NS@CC is evaluated by galvanostatic charging (Fig. S12), which shows the areal capacity of ~ 60 mAh cm^−2^. Figure S13 also shows the strong reducibility of molten Li, which can convert Ni_5_P_4_ into Li_3_P and Ni metal.

To prove the excellent stability of the Li-Ni/Li_m_X-NS@CC electrode (taking the Li-Ni/Li_3_N-NS@CC as an example*), *in situ XRD was performed to observe the phase change during the stripping/plating process. The in situ XRD patterns (Fig. 2e–f) show that no new phase is formed and the peaks of Ni metal do not change during the electrochemical deposition and dissolution process. The Li_3_N peaks are not detected, which should be masked by stronger Be peaks. Furthermore, the ex situ XRD reveals that the Li_3_N phase remains after 20 cycles and different stripping capacities (Figs. S14 and S15), demonstrating that the Li_3_N phase is very stable during electrochemical cycles. Therefore, the Li_m_X phases will not change during the electrochemical stripping and plating process, which is beneficial for regulating Li deposition/dissolution.

### Chemical Adsorption/Diffusion Regulation Analysis

**Fig. 3:**
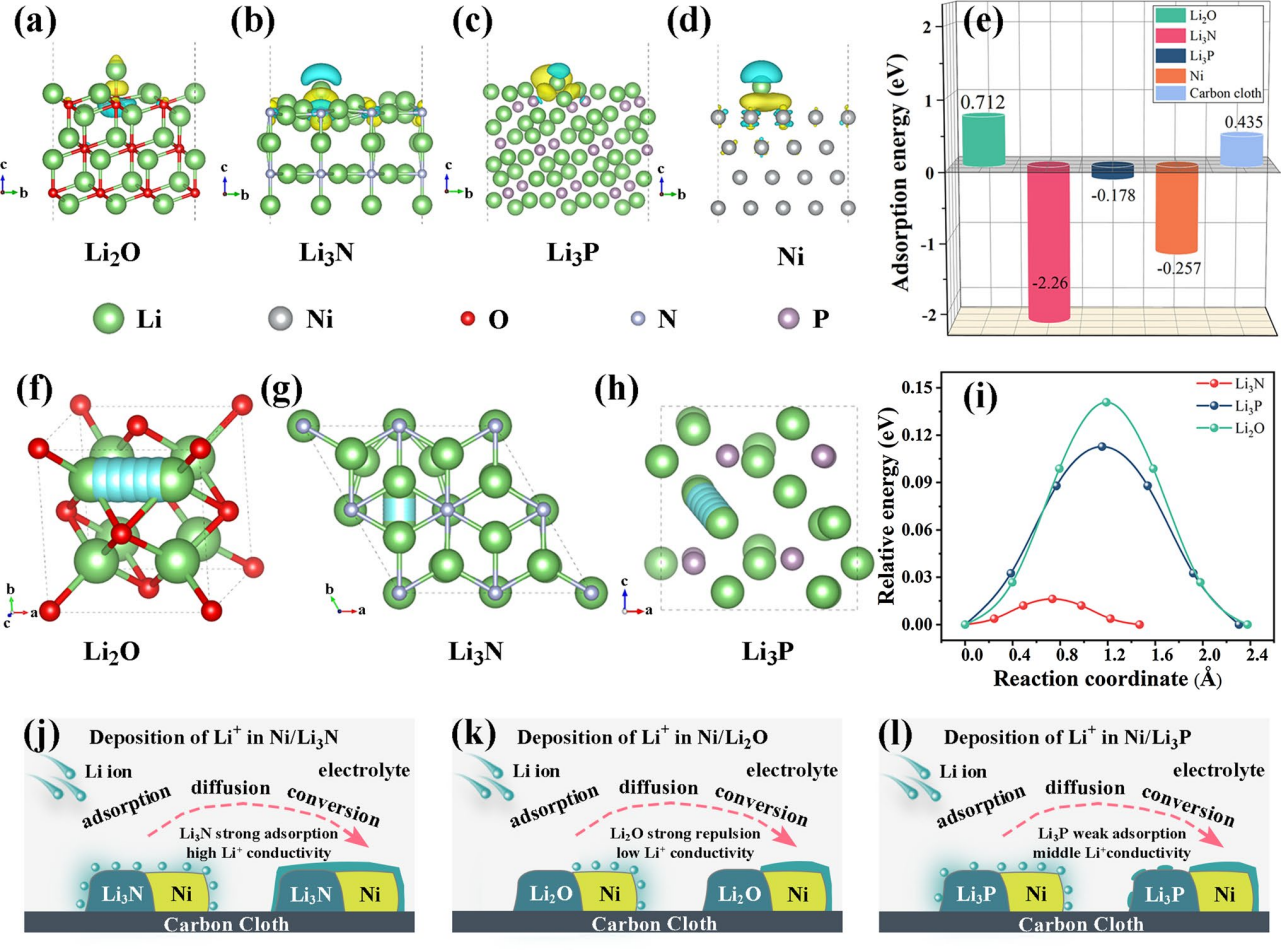
Adsorption model structure and differential charge density of Li atom on **a** Li_2_O, **b** Li_3_N, **c** Li_3_P, and **d** Ni. The yellow and cyan regions represent electron accumulation and depletion, respectively. **e** The corresponding adsorption energy of a Li atom on crystal surfaces. The optimized diffusion path through **f** Li_2_O, **g** Li_3_N, and **h** Li_3_P. **i** The comparison plot of the corresponding energy barrier. Illustrations of the working mechanism of **j** Ni/Li_3_N, **k** Ni/Li_2_O, and **l** Ni/Li_3_P for lithium ion deposition

DFT calculations were further conducted to reveal the chemical regulation effect of Ni/Li_m_X on lithium adsorption and diffusion. The adsorption energies (Figs. 3a–e and S16) of a Li atom absorbed on Li_2_O, Li_3_N, Li_3_P, Ni, and carbon cloth are 0.712, − 2.26, − 0.178, − 1.257, and 0.435 eV, respectively. The Li^+^ diffusion energy barriers in Li_2_O, Li_3_N, and Li_3_P crystals were also evaluated (Figs. 3f–h and S17–S19). The optimized Li^+^ diffusion paths in Li_2_O, Li_3_N, and Li_3_P crystals are illustrated in Fig. 3f–h, and the corresponding diffusion energy barriers (Fig. 3i) are estimated to be 0.141, 0.016, and 0.113 eV, respectively. Therefore, the difference in the electrochemical behavior of Ni/Li_m_X nanosheets decorated on carbon cloth (schematically shown in Fig. S20) toward Li plating is summarized in Fig. 2j–l. Since the adsorption energy of Li_3_N and Ni to Li atom is larger than that of Li_2_O and Li_3_P, the Li ions are uniformly distributed around the Ni/Li_3_N nanosheets (Fig. 2j). In addition, the high Li^+^ conductivity in Li_3_N is also conducive to achieving the uniform allocation of Li^+^ on the electrode surface [42, 69]. Contrastingly, Li_2_O provides the strongest repulsion to Li atom and the lowest Li^+^ conductivity, resulting in uneven Li deposition in the Li-Ni/Li_2_O-NS@CC electrode (Fig. 2k). Li_3_P delivers middle Li^+^ conductivity and adsorption energy, which also leads to rough Li deposition in the Li-Ni/Li_3_P-NS@CC electrode (Fig. 2l). Therefore, it is theoretically predicted that the Li-Ni/Li_3_N-NS@CC electrode with high ionic conductivity and strong lithiophilicity is beneficial to suppressing dendrite growth and stabilizing Li metal anodes.

### Electrochemical Performance of the Li-Ni/Li_m_X-NS@CC Electrodes

**Fig. 4:**
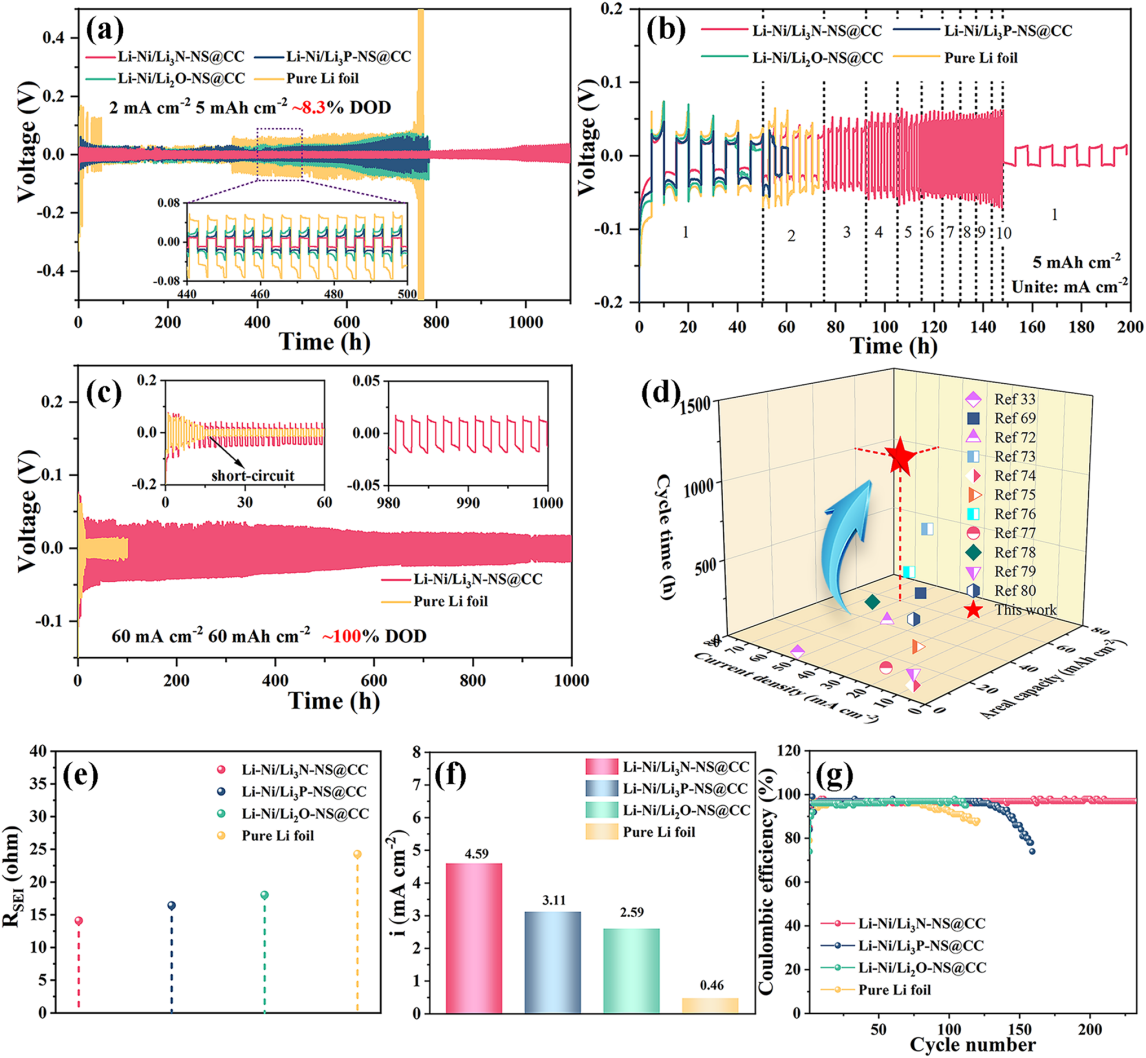
**a** Voltage–time profiles of symmetrical batteries with the Li-Ni/Li_2_O-NS@CC, the Li-Ni/Li_3_N-NS@CC, the Li-Ni/Li_3_P-NS@CC, and pure Li electrodes at 2 mA cm^−2^, 5 mAh cm^−2^. **b** The rate performance of the above electrodes obtained at different current densities, respectively. **c** The voltage–time profiles of symmetrical batteries with the Li-Ni/Li_3_N-NS@CC and pure Li electrodes at 60 mA cm^−2^, 60 mAh cm^−2^. **d** Long-cycle performance comparison of symmetrical battery with the Li-Ni/Li_3_N-NS@CC electrode and other published works recently. **e** The comparison of *R*_SEI_ of the above electrodes after cycling. **f** The comparison of exchanging current density among various samples. **g** A comparison of CE of Li||Cu batteries based on the Li-Ni/Li_2_O-NS@CC, the Li-Ni/Li_3_N-NS@CC, the Li-Ni/Li_3_P-NS@CC and pure Li electrodes at 1 mA cm^−2^, 1 mAh cm^−2^ (counter electrode: Cu foil)

To examine the electrochemical stability of the Li-Ni/Li_m_X-NS@CC electrodes, symmetrical batteries are assembled using an ether-based electrolyte. Obviously, at the current density of 1.0 mA cm^−2^ and the capacity of 1.0 mAh cm^−2^, the Li-Ni/Li_3_N-NS@CC symmetrical battery displays stable and ultralong cycling for 2000 h with an admirable small overpotential of 19.4 mV (Fig. S21). In contrast, the pure Li symmetrical battery exhibits sharp voltage fluctuation and a high overpotential of 113 mV after 100 h, which can be attributed to the unstable SEI layer and continuous side reaction [70, 71]. The symmetrical batteries with the Li-Ni/Li_2_O-NS@CC and the Li-Ni/Li_3_P-NS@CC electrode can also deliver stable cycling, respectively, but their voltage hysteresis (39.9 mV for Li-Ni/Li_2_O-NS@CC electrode and 33.4 mV for Li-Ni/Li_3_P-NS@CC electrode) are relatively higher than that of the Li-Ni/Li_3_N-NS@CC. In addition to ether-based electrolytes, the Li-Ni/Li_3_N-NS@CC symmetrical battery can also achieve stable cycling performance in carbonate-based electrolytes at 1 mA cm^−2^ and 1 mAh cm^−2^ (Fig. S22). Compared with pure Li symmetric battery, the smaller overpotential of Li-Ni/Li_3_N-NS@CC symmetric battery in carbonate electrolyte indicates more uniform Li stripping/plating on Li-Ni/Li_3_N-NS@CC electrode. As the current density is increased to 2 mA cm^−2^ and the areal capacity is raised to 5 mAh cm^−2^ (Fig. 4a), the Li-Ni/Li_3_N-NS@CC symmetrical battery still shows a small overpotential (~ 21.9 mV) for 1100 h. Conversely, the pure Li symmetrical battery exhibits serious voltage oscillation after 340 h. The overpotential of the Li-Ni/Li_3_P-NS@CC and the Li-Ni/Li_2_O-NS@CC symmetrical cells begins to increase after cycling for 400 h possibly due to the low Li^+^ conductivity and unsuitable adsorption energy. The Li@CC symmetrical battery (Fig. S23) exhibits short circuit after 40 h, indicating that both morphology and composition are effective for stable Li plating and stripping. Furthermore, the symmetric battery with the Li-Ni/Li_3_N-NS@CC electrode displays superior rate performance under increasing current densities up to 10 mA cm^−2^ with 5 mAh cm^−2^ (overpotential ≈ 80 mV, Fig. 4b), while the symmetrical batteries with other electrodes suffer from a short circuit at the low current density of 2 mA cm^−2^. Figure 4c shows that the symmetrical battery with the Li-Ni/Li_3_N-NS@CC electrode impressively displays small overpotential and long-term stability for 1000 h at ultrahigh current density (60 mA cm^−2^) and areal capacity (60 mAh cm^−2^). Meanwhile, the batteries with pure Li show poor cyclability and become short circuit at 60 mA cm^−2^ (~ 10 h for pure Li electrode), indicating dendrites pierce the separator. Importantly, such an outstanding long-cycle performance (60 mA cm^−2^, 60 mAh cm^−2^) greatly exceeds that of the most published Li metal anodes, as shown in Fig. 4d [33, 69, 72–80].

The lowest hysteresis of the Li-Ni/Li_3_N-NS@CC symmetrical cell is mainly ascribed to the excellent lithiophilicity, and fast mass migration, which can also be affirmed by electrochemical impedance spectroscopy measurements (EIS) (Figs. 4e and S24). The SEI resistance (*R*_SEI_) and charge transfer resistance (*R*_ct_) of the symmetric batteries are analyzed using the equivalent circuit in Fig. S25. After 20 cycles, the *R*_SEI_ of the Li-Ni/Li_3_N-NS@CC anode maintains the lowest, facilitating fast mass migration and dendrite‐free plating/stripping in the Li-Ni/Li_3_N-NS@CC electrode [47, 81, 82]. The *R*_ct_ (Fig. S26) of the Li-Ni/Li_3_N-NS@CC anode still maintains the lowest value due to the lowest Li^+^ diffusion energy barrier of Li_3_N [83]. Exchange current density (*I*_0_) obtained from the Tafel plot can reflect charge transfer kinetics between the electrode and electrolyte components. Based on Tafel plots in Fig. S27, the calculated results (Fig. 4f) show that the exchange current density of the Li-Ni/Li_3_N-NS@CC (*I*_0_ = 4.59 mA cm^−2^) is the largest than that of the Li-Ni/Li_3_P-NS@CC (*I*_0_ = 3.11 mA cm^−2^), the Li-Ni/Li_2_O-NS@CC (*I*_0_ = 2.59 mA cm^−2^) and the pure Li electrode (*I*_0_ = 0.46 mA cm^−2^), demonstrating that the SEI on the Li-Ni/Li_3_N-NS@CC electrode can drastically achieve fast Li^+^ transfer kinetics. Figures 4g and S28 exhibit the CE of various electrodes and typical charge–discharge curves, respectively. The CE value for pure Li foil slips sharply after 80 cycles, while the battery with the Li-Ni/Li_3_N-NS@CC electrode maintains a high CE value (97.6%) for over 225 cycles (Fig. 4g). To further prove the superiority of the Li-Ni/Li_3_N-NS@CC electrode, the CE of the Li-Ni/Li_3_P-NS@CC and the Li-Ni/Li_2_O-NS@CC electrodes was also investigated, which starts to drop after 100 cycles for the Li-Ni/Li_2_O-NS@CC electrode and after 125 cycles for the Li-Ni/Li_3_P-NS@CC electrode. Figure S28 exhibits that the voltage curves of Li plating/stripping for the second cycle. The voltage hysteresis of the Li-Ni/Li_3_N-NS@CC electrode (26 mV) is much smaller than that for the Li-Ni/Li_3_P-NS@CC electrode (34 mV) and Li-Ni/Li_2_O-NS@CC electrode (57 mV), which can be ascribed to the appropriate Li binding energy of Li_3_N/Ni. These results may lead to the ultra-flat deposition of Li, further inhibiting the overgrowth of SEI during the long-term cycle [3, 84]. Overall, the Li-Ni/Li_3_N-NS@CC electrode affords the advantages of the least resistance, the highest exchange current density and the lowest Li voltage hysteresis, indicating its great potentiality as Li hosts to inhibit Li dendrite formation and improve Li metal battery performances.

### Morphology Evolutions of the Li-Ni/Li_3_N-NS@CC Electrode

**Fig. 5:**
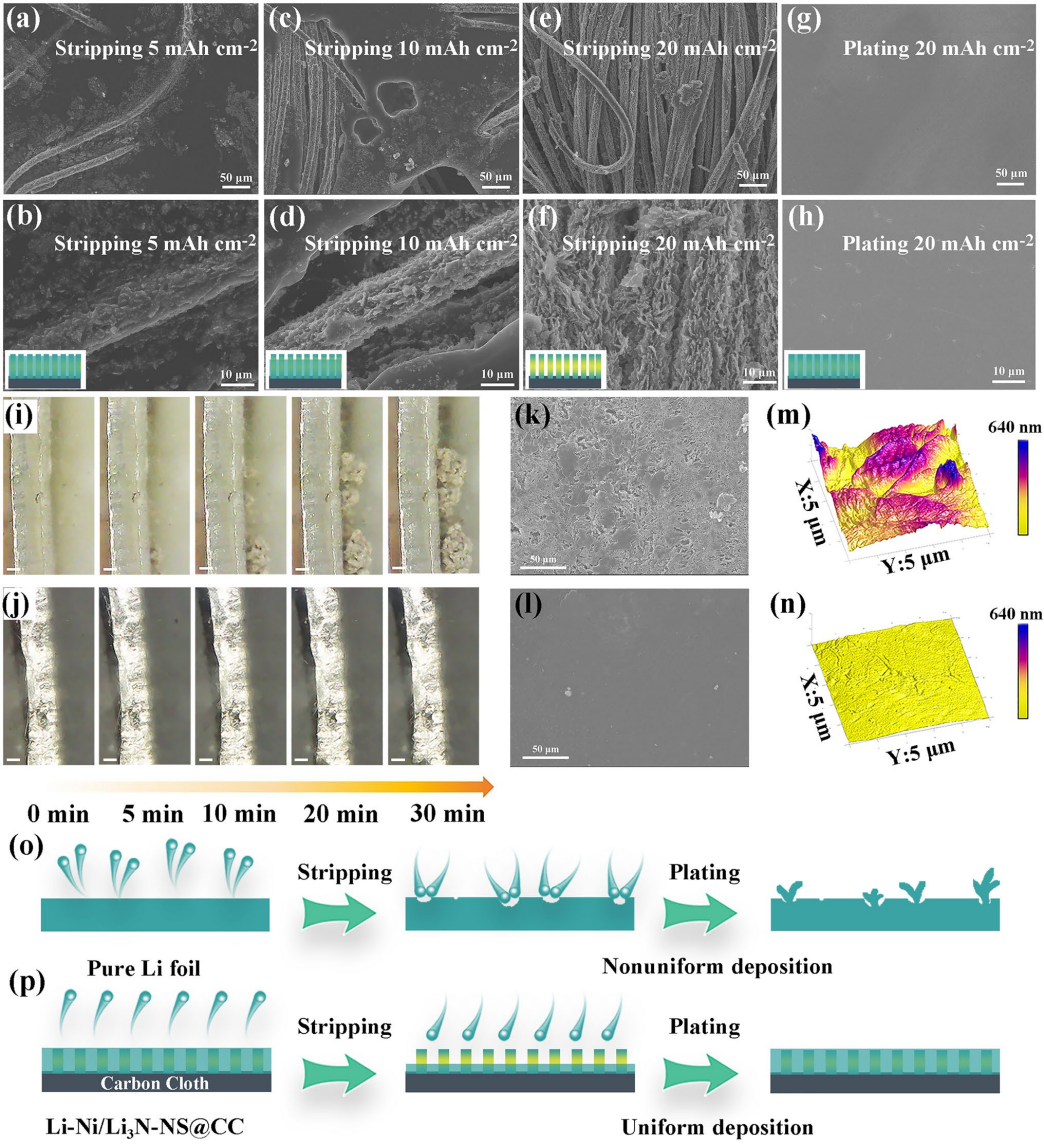
SEM images of the Li-Ni/Li_3_N-NS@CC electrode after stripping **a**, **b** 5 mAh cm^−2^, **c**, **d** 10 mAh cm^−2^, **e**, **f** 20 mAh cm^−2^ and replating **g**, **h** 20 mAh cm^−2^. Operando observation of the Li plating on **i** pure Li and **j** Li-Ni/Li_3_N-NS@CC electrode and the scale bar is 100 µm. SEM images of **k** pure Li and **l** Li-Ni/Li_3_N-NS@CC electrodes after 20 cycles at 5 mA cm^−2^ and 5 mAh cm^−2^. AFM images of **m** pure Li and **n** the Li-Ni/Li_3_N-NS@CC electrodes after cycling, respectively. Schematic illustration of the Li stripping/plating behavior of **o** pure Li foil and **p** the Li-Ni/Li_3_N-NS@CC electrodes

The morphology evolutions of the Li-Ni/Li_3_N-NS@CC electrode during the process of stripping/plating under various areal capacities are intuitively explored. When 5 mAh cm^−2^ is stripped, a small part of carbon fibers is exposed in the Li-Ni/Li_3_N-NS@CC electrode (Fig. 5a), revealing that the stripping process of lithium metal is from top to bottom. The surface morphology of carbon fibers (Fig. 5b) shows that the nanosheets are fully infiltrated with Li metal. With the stripping capacity increased to 10 mAh cm^−2^, more carbon fibers are manifested (Fig. 5c–d). When Li stripping capacity is further raised to 20 mAh cm^−2^, the nanosheet array (Fig. 5e–f) becomes evident, indicating the marvelous structure stability. The SEM images (Fig. S29) of Li-Ni/Li_2_O-NS@CC and Li-Ni/Li_3_P-NS@CC electrodes after stripping 20 mAh cm^−2^ exhibit uneven Li exfoliation morphologies, demonstrating Li_3_N with high ion conductivity is conducive to uniform exfoliation of lithium metal [38]. Subsequently, 20 mAh cm^−2^ of Li metal is redeposited onto the Li-Ni/Li_3_N-NS@CC electrode, and the smooth and flat surface without Li dendrite can be observed (Fig. 5g–h), which is assigned to uniform nucleation and fast ion migration. The stripping/plating process of the Li-Ni/Li_3_N-NS@CC is thus schematically illustrated by the insets in Fig. 5b, d, f, h. Furthermore, the in situ process of Li^+^ plating in the Li-Ni/Li_3_N-NS@CC and pure Li electrodes was recorded by operando optical microscopy. For pure Li electrodes, small Li dendrites start to grow on the surface of the pure Li electrode after only 5 min (Fig. 5i). After plating for 30 min, the Li dendrites are overflowed at the sides of the pure Li electrode. While the surface of the Li-Ni/Li_3_N-NS@CC electrode remains smooth and dendrite-free morphology is discerned during the whole deposition process (Fig. 5j). In Fig. 5k, dendritic Li materializes on pure Li electrode after 20 plating/stripping cycles at 5 mA cm^−2^ with a specific capacity of 5 mAh cm^−2^. Contrastingly, the smooth top-view morphology of the Li-Ni/Li_3_N-NS@CC electrode is sustained when tested under the same condition (Fig. 5l). AFM image shows that the top surface (Fig. 5m) of pure Li is rough, while the Li-Ni/Li_3_N-NS@CC electrode (Fig. 5n) has a smooth and uniform surface. Figure 5o–p schematically summarize the Li^+^ deposition/dissolution behavior of pure Li foil and the Li-Ni/Li_3_N-NS@CC electrodes, respectively. The Li-Ni/Li_3_N-NS@CC electrode with uniform Li^+^ flow, fast ion transfer kinetics of Li_3_N, and abundant lithiophilic sites displays uniform lithium stripping/plating on the carbon fibers and dendrite-free morphology. Contrastingly, for pure Li electrodes, Li^+^ prefers to deposit in pits and Li^+^ localized deposition drives massive Li dendrite growth, impeding the cyclic stability of LMA.

### Applicability of the Li-Ni/Li_3_N-NS@CC Electrode in Full Battery

**Fig. 6:**
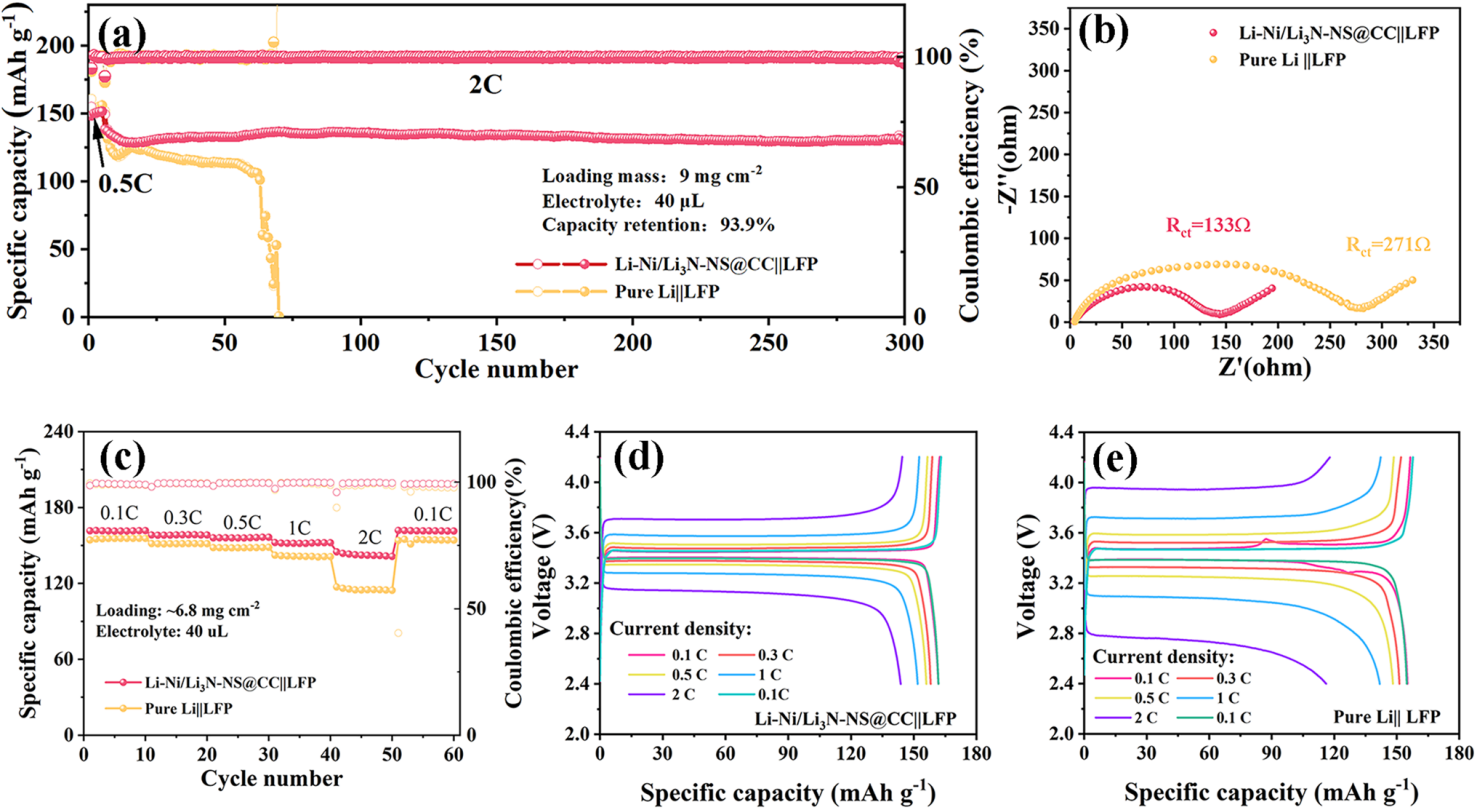
**a** Cycling stability obtained at a current density of 2C with high loading mass of 9 mg cm^−2^. **b** The Nyquist plots of full batteries with the Li-Ni/Li_3_N-NS@CC, and pure Li electrodes after cycling. **c** Rate performance measured from 0.1 to 2C. Galvanostatic charge–discharge profiles of **d** Li-Ni/Li_3_N-NS@CC||LFP and **e** pure Li||LFP full batteries obtained at different current densities

To highlight the applicability of the Li-Ni/Li_3_N-NS@CC electrode, the cyclic stability (Fig. 6a) of full batteries coupled with LiFePO_4_ (LFP) cathode was conducted at 2C (1 C = 170 mA g^−1^). It is noted that the mass loading of LFP is about 9 mg cm^−2^ and the fixed amount of electrolyte used in each cell is 40 µL. Significantly, Li-Ni/Li_3_N-NS@CC||LFP battery demonstrates stable cycling performance with 93.9% retention after 300 cycles, which exceeds that of the pure Li||LFP battery (decaying rapidly after 60 cycles). Porous Li dendrite (Fig. S30a) is observed on pure Li foil after 40 cycles in full battery, while a compact and smooth Li (Fig. S30b) strips/plats on Li-Ni/Li_3_N-NS@CC electrode. The typical voltage profile (Fig. S31) of the Li-Ni/Li_3_N-NS@CC||LFP battery shows a smaller polarization voltage (460 mV) during the 10th cycle than that (787 mV) of the pure Li||LFP battery. Figure 6b displays that the *R*_ct_ value (133 Ω) of the Li-Ni/Li_3_N-NS@CC||LFP battery is lower than that (271 Ω) of the pure Li||LFP battery, illustrating fast Li^+^ migration in the Li-Ni/Li_3_N-NS@CC electrode. Moreover, the Li-Ni/Li_3_N-NS@CC||LFP battery reveals a better rate capability than that of the pure Li||LFP battery (Fig. 6c). Specifically, the discharge capacities of the Li-Ni/Li_3_N-NS@CC||LFP battery are 161, 158, 156, 151, and 142 mAh g^−1^ at 0.1, 0.3, 0.5, 1, and 2 C respectively, higher than those of pure Li||LFP (155, 151, 148, 141, and 115 mAh g^−1^, shown in Fig. 6d–e). These results indicate that Li-Ni/Li_3_N-NS@CC electrode enables Li metal batteries with significantly enhanced cyclic stability and rate performance.

## Conclusions

Physical morphology confinement and chemical adsorption/diffusion regulation have been proposed to optimize Ni-based compounds decorated carbon cloth as the host for Li metal anodes. Finite element simulations prove that compared with 1D nanowires and 0D nanoparticles, the 2D nanosheets deliver the best physical morphology confinement effect. Furthermore, DFT calculations and in/ex situ experimental results demonstrate that Li_3_N with the strongest adsorption energy and the lowest diffusion energy barrier can facilitate the uniform Li^+^ flux and manipulate the uniform Li^+^ nucleation. As a result, the Li-Ni/Li_3_N-NS@CC electrode can realize uniform Li^+^ nucleation and a long lifespan (1000 h) at 60 mA cm^−2^/60 mAh cm^−2^ with a dendrite-free morphology. Moreover, the full battery paired with the Li-Ni/Li_3_N-NS@CC electrode displays better cycle stability than the full battery with a pure Li anode. The emphasis of this work is on optimizing Ni-based lithiophilic compounds for protecting Li metal anode. The combined experimental and computational approach used in this work is also extensively appropriate to exploit other lithiophilic compounds for LMA systems.

## Supplementary Information

Below is the link to the electronic supplementary material.

Supplementary file1 (PDF 1444 KB)
