# Supplementary material for: Revisiting the Role of Physical Confinement and Chemical Regulation of 3D Hosts for Dendrite-Free Li Metal Anode
Source: Nanomicro Lett. 2022 Sep 14;14:187. doi: 10.1007/s40820-022-00932-3 (PMC9474970; doi:10.1007/s40820-022-00932-3)
Supplement: Supplementary file 1 — Supplementary file1 (PDF 1444 KB) [file 40820_2022_932_MOESM1_ESM.pdf]

Supporting Information for

Revisiting the Role of Physical Confinement and Chemical Regulation of 3D Hosts for Dendrite-Free Li Metal Anode

Shufen Ye[1, #], Xingjia Chen[1, #], Rui Zhang[2, #], Yu Jiang[3], Fanyang Huang[1], Huijuan Huang[1], Yu Yao[1], Shuhong Jiao[1], Xiang Chen[4], Qiang Zhang[4, *], Yan Yu[1, 5, *]

[1]Hefei National Center for Physical Sciences at the Microscale, Department of Materials Science and Engineering, *i*ChEM (Collaborative Innovation Center of Chemistry for Energy Materials), CAS Key Laboratory of Materials for Energy Conversion, University of Science and Technology of China, Hefei, Anhui 230026, P. R. China

[2]Advanced Research Institute of Multidisciplinary Science, Beijing Institute of Technology, Beijing 100081, P. R. China

[3]School of Materials Science and Engineering, Anhui University, Hefei, Anhui 230601, P. R. China

[4]Beijing Key Laboratory of Green Chemical Reaction Engineering and Technology, Department of Chemical Engineering, Tsinghua University, Beijing 100084, P. R. China

[5]National Synchrotron Radiation Laboratory, Hefei, Anhui 230026, P. R. China

[#]Shufen Ye, Xingjia Chen, and Rui Zhang contributed equally to this work.

*Corresponding authors. E-mail: yanyumse@ustc.edu.cn (Yan Yu); zhang-qiang@mails.tsinghua.edu.cn (Qiang Zhang)

Supplementary Figures and Table

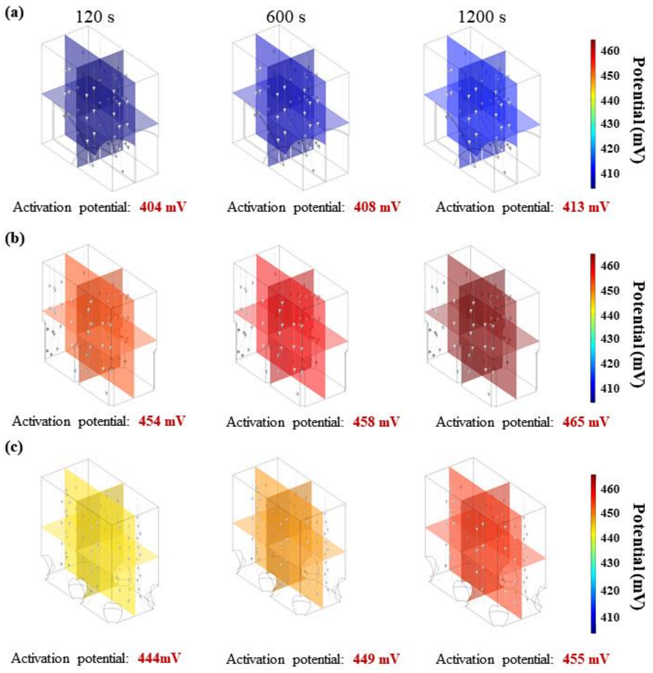

Fig. S1 a The simulated electrolyte electric potential and current line near carbon cloth with 2D nanosheets during Li electroplating after 120, 600 and 1200 s. **b** The simulated electrolyte electric potential and current line near carbon cloth with 1D nanowires during Li electroplating after 120, 600, and 1200 s. **c** The simulated electrode electric potential and current line near carbon cloth with 0D nanoparticles during Li electroplating after 120, 600 and 1200 s

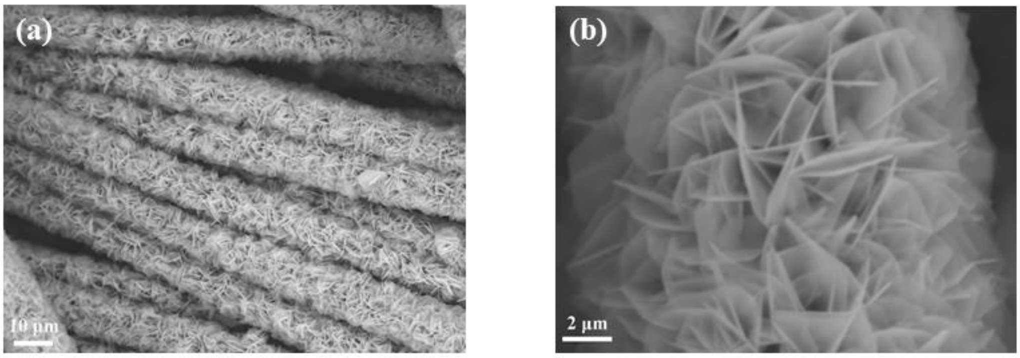

Fig. S2 a The top-view and **b** enlarged top-view SEM images of Ni precursors

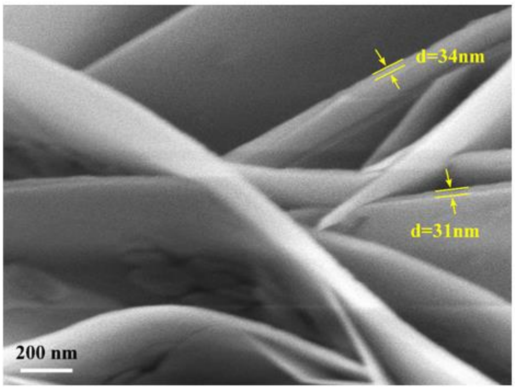

Fig. S3 Enlarged top-view SEM images of Ni precursor. The thickness of the nanosheet is about 30 nm

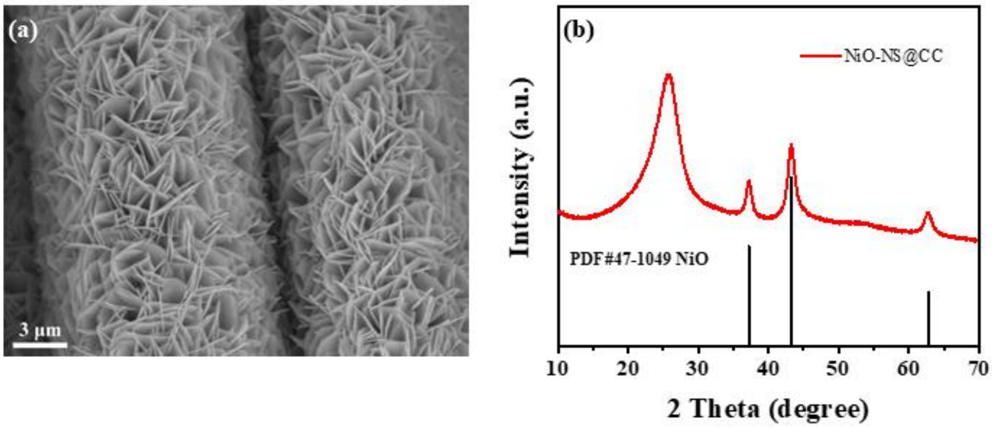

Fig. S4 a The top-view SEM image and **b** XRD pattern of the NiO-NS@CC

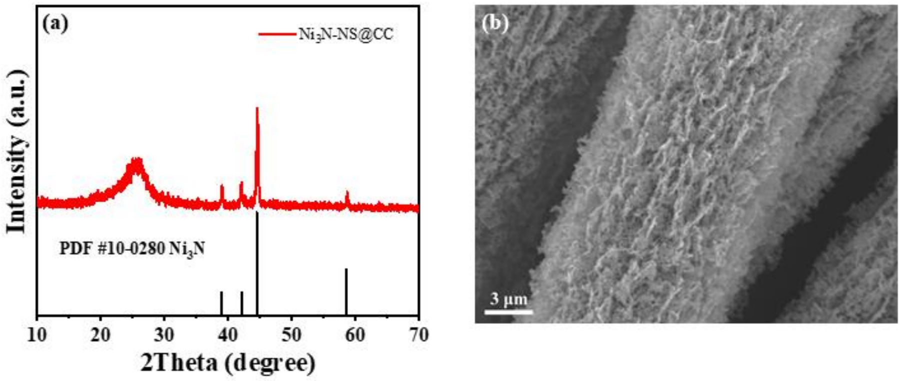

Fig. S5 a XRD pattern and **b** top-view SEM image of the Ni_3N-NS@CC

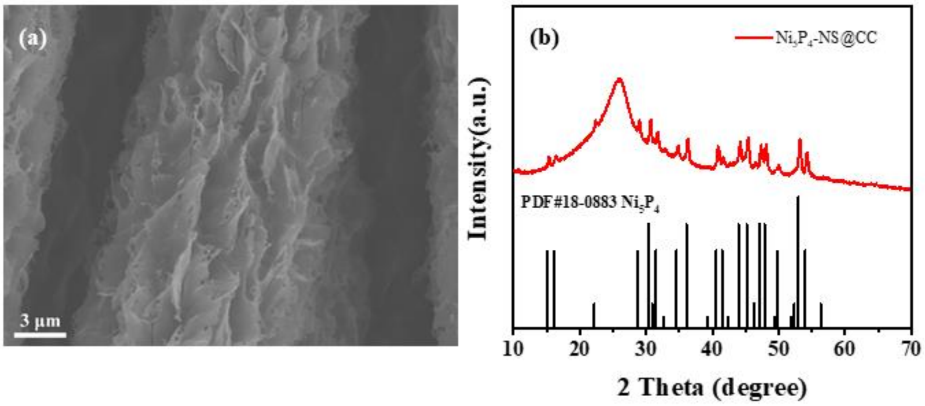

Fig. S6 a The top-view SEM image and **b** XRD pattern of the Ni_5P_4-NS@CC

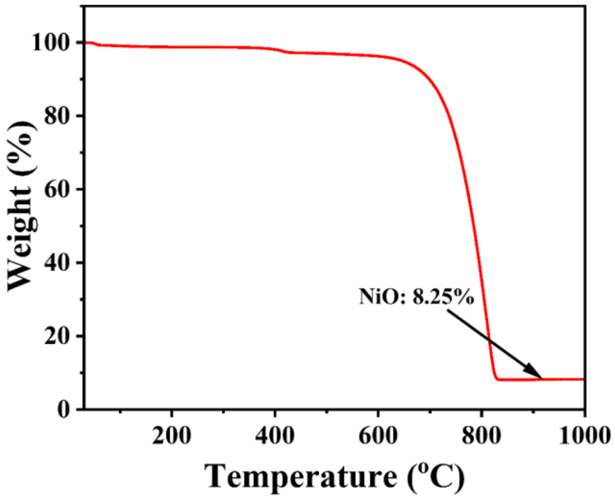

Fig. S7 TGA curve of Ni-precursor-NS@CC

The content of Ni element ([Ni]) can be calculated by element quality conversation during TGA test. Ni-precursor-NS completely transformed into the pure NiO.

$$[Ni]=59*8.25\%/75=6.49\%$$

It is assumed that the content of Ni element remains constant during the oxidation, nitridation and phosphating process of Ni-precursor-NS@CC. The loading amount of NiO, Ni_3N, and Ni_5P_4 as following:

$$[NiO]= 8.25\%$$

$$[Ni_3N]\approx 7.09\%$$

$$[Ni_5P_4]\approx 8.93\%$$

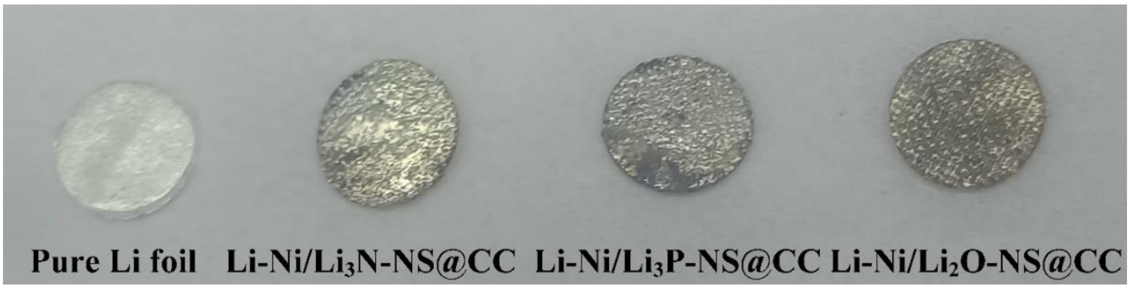

Fig. S8 The digital photos of pure Li foil, Li-Ni/Li_3N-NS@CC, Li-Ni/Li_3P-NS@CC, and Li-Ni/Li_2O-NS@CC

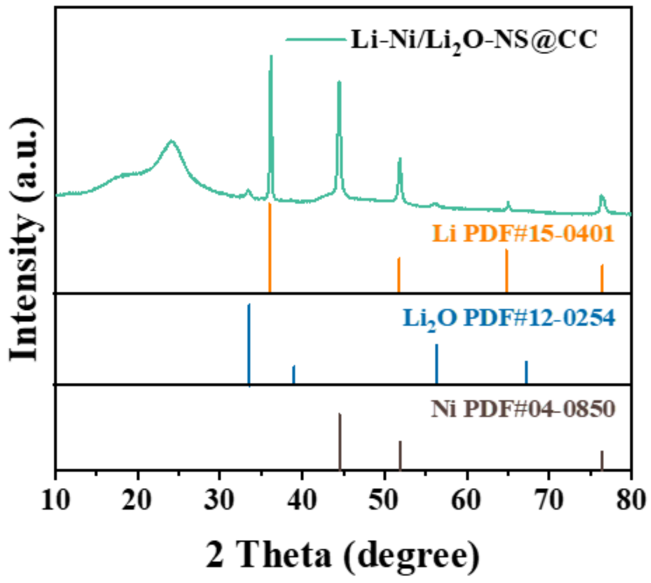

Fig. S9 XRD pattern of the Li-Ni/Li_2O-NS@CC

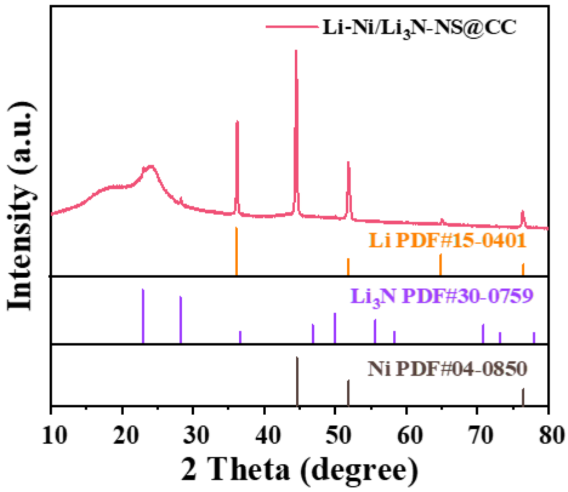

Fig. S10 XRD pattern of the Li-Ni/Li_3N-NS@CC

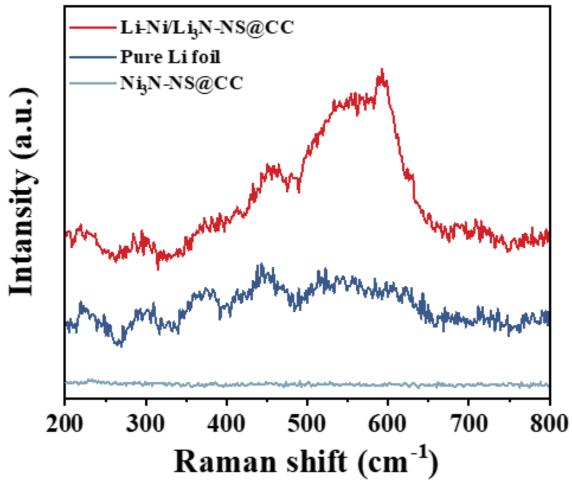

Fig. S11 The comparison of Raman spectra of the Ni_3N-NS@CC, pure Li, and the Li-Ni/Li_3N-NS@CC

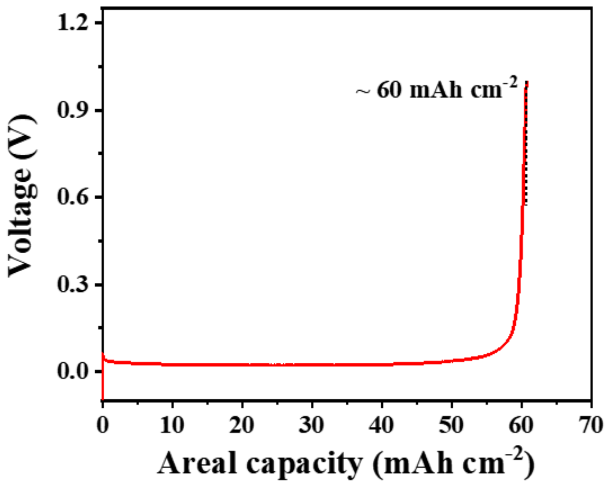

Fig. S12 Full Li stripping curve of the Li-Ni/Li_3N-NS@CC electrode to 1 V *vs* Li^+/Li

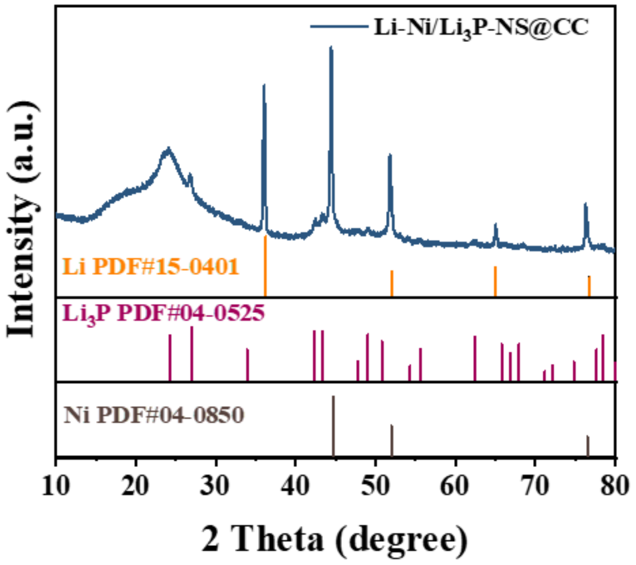

Fig. S13 XRD pattern of the Li-Ni/Li_3P-NS@CC

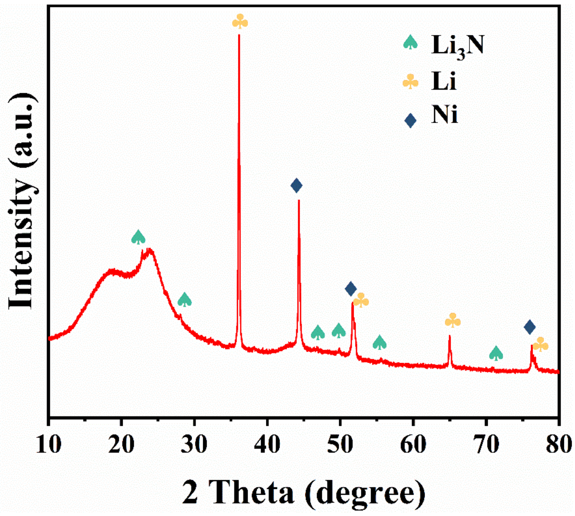

Fig. S14 XRD pattern of the Li-Ni/Li_3N-NS@CC electrode after 20 cycles at 1 mA cm^{-2} and 1 mAh cm^{-2}

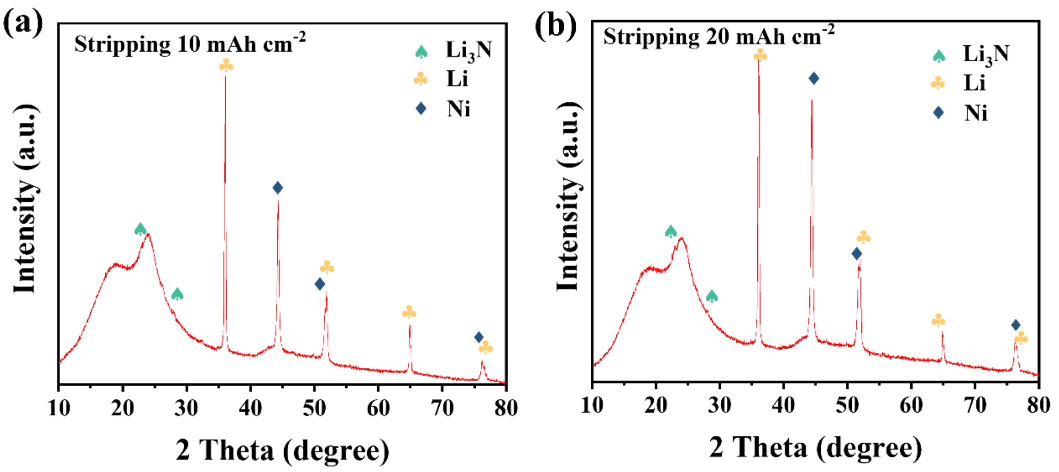

Fig. S15 XRD patterns of the Li-Ni/Li_3N-NS@CC electrode after stripping **a** 10 mAh cm^{-2} and **b** 20 mAh cm^{-2}

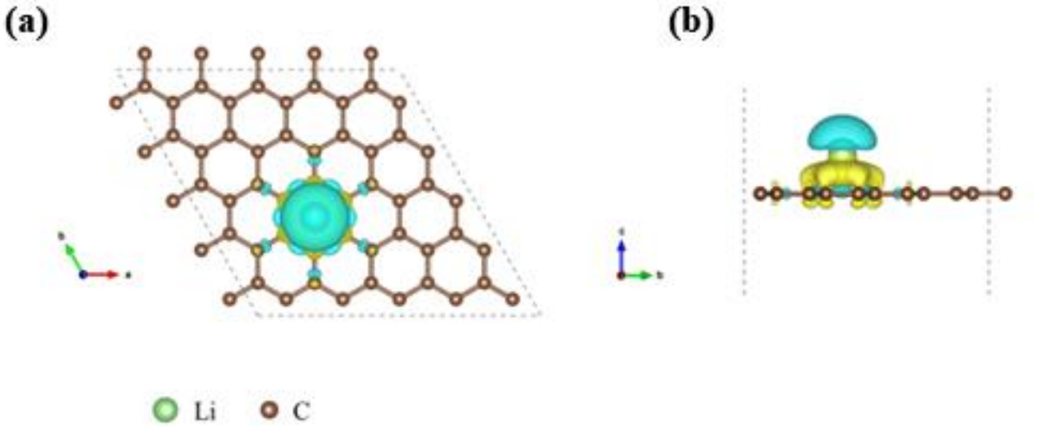

Fig. S16 Adsorption model structure and electron cloud density of Li atom on carbon cloth. The adsorption energies of a Li atom absorbed carbon cloth is 0.435 eV

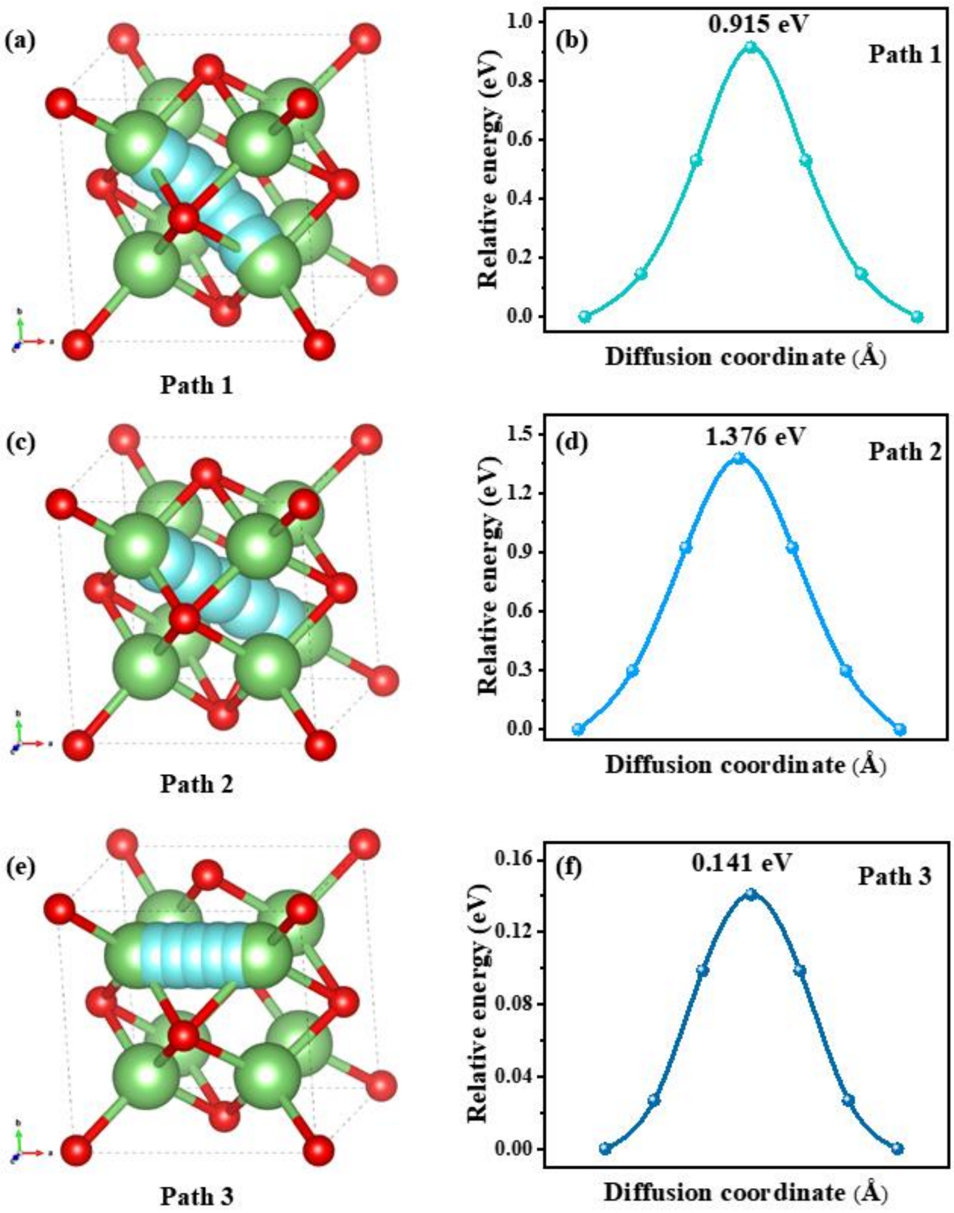

Fig. S17 The simulated three migration pathways of a Li atom and the corresponding energy barrier through the Li_2O crystal

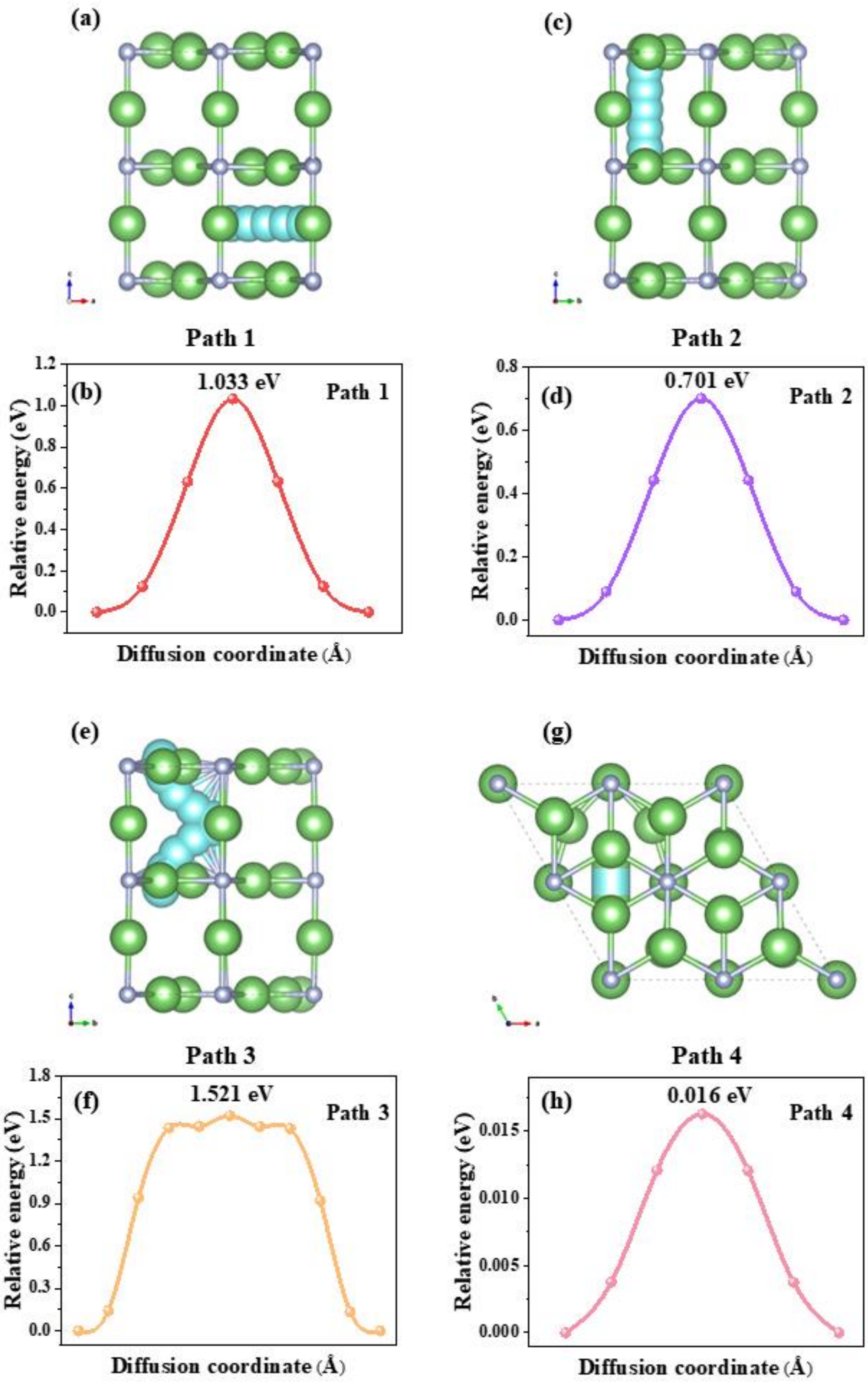

Fig. S18 The simulated four migration pathways of a Li atom and the corresponding energy barrier through the Li_3N crystal

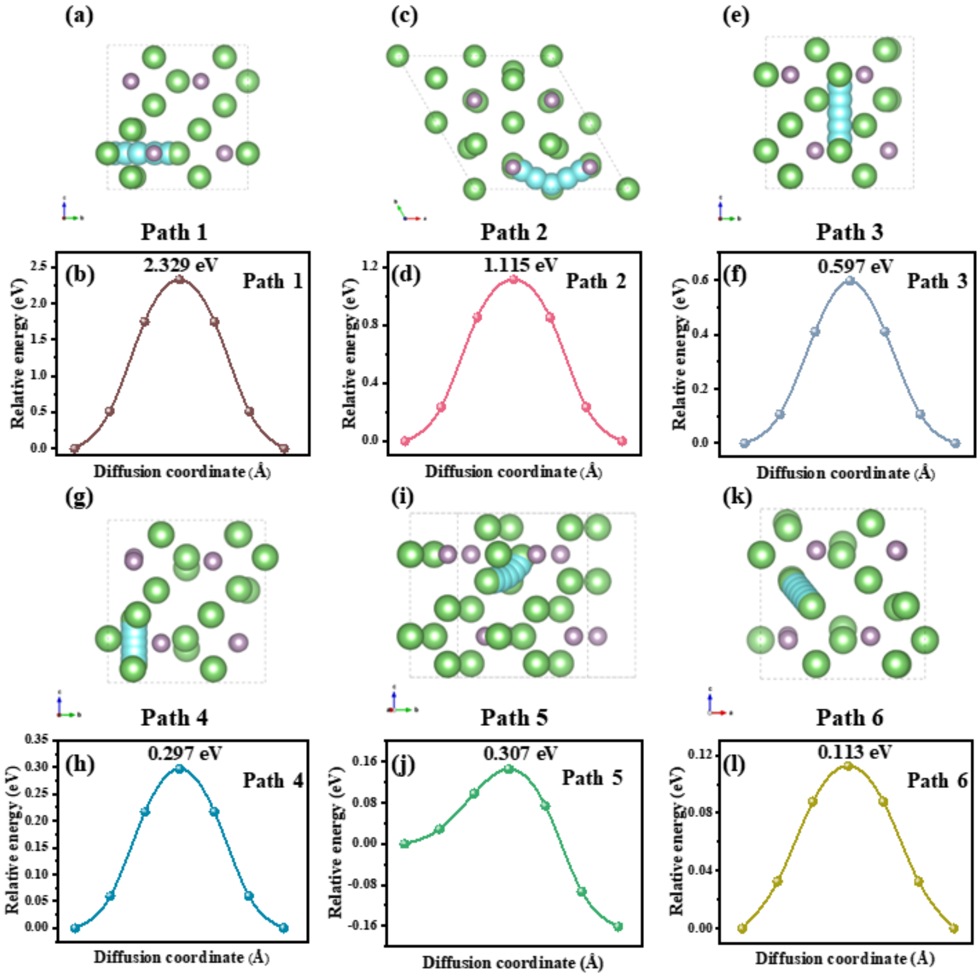

Fig. S19 The simulated six migration pathways of a Li atom and the corresponding energy barrier through the Li_3P crystal

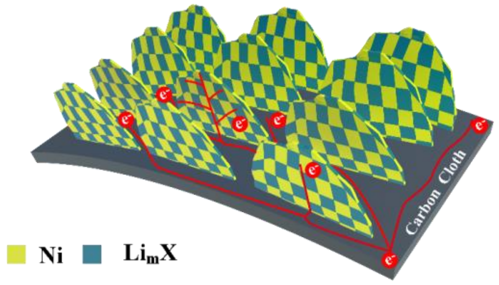

Fig. S20 Schematic illustration of the structure of the Ni/Li_mX-NS@CC

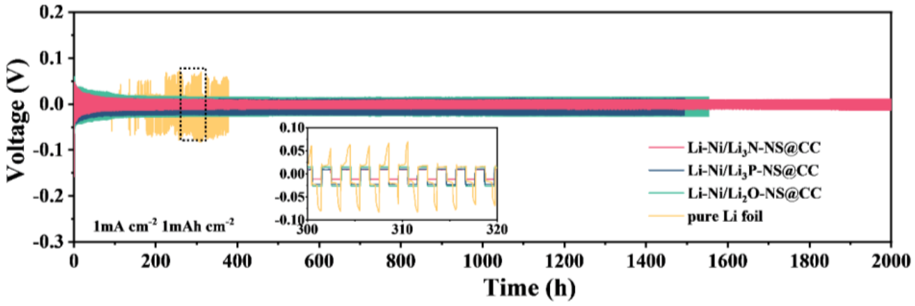

Fig. S21 Voltage–time profiles of symmetrical batteries with the Li-Ni/Li_2O-NS@CC, the Li-Ni/Li_3N-NS@CC, the Li-Ni/Li_3P-NS@CC and pure Li electrodes at 1 mA cm^{-2} and 1 mAh cm^{-2}

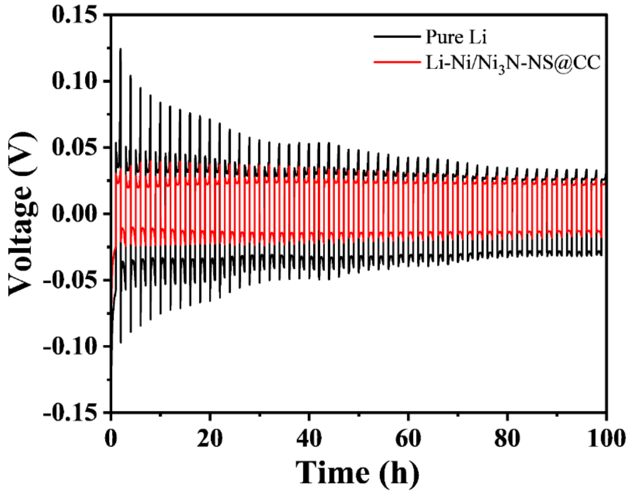

Fig. S22 Voltage–time profiles of symmetrical batteries with the Li-Ni/Li_3N-NS@CC and pure Li electrodes at 1 mA cm^{-2} and 1 mAh cm^{-2} in carbonate electrolyte

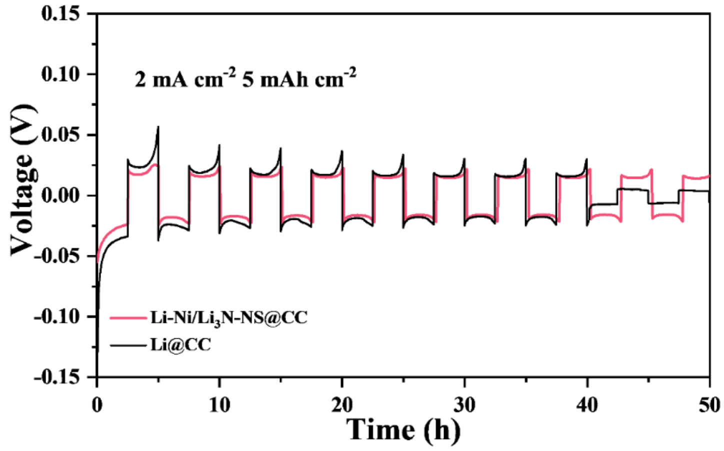

Fig. S23 Voltage–time profiles of symmetrical batteries with the Li-Ni/Li_3N-NS@CC and Li@CC electrodes at 2 mA cm^{-2} and 5 mAh cm^{-2} in ether-based electrolyte

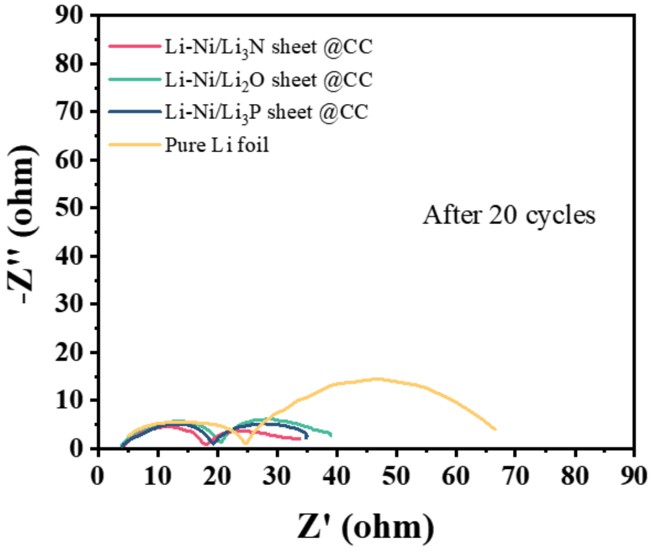

Fig. S24 The Nyquist plots of symmetrical batteries with the Li-Ni/Li_2O-NS@CC, the Li-Ni/Li_3N-NS@CC, the Li-Ni/Li_3P-NS@CC, and pure Li electrodes after cycling

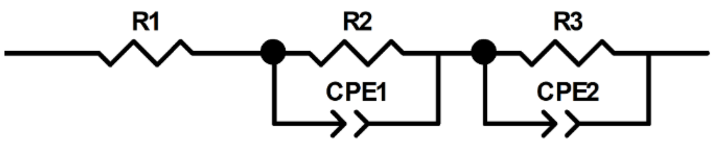

Fig. S25 The equivalent circuit of Nyquist plot

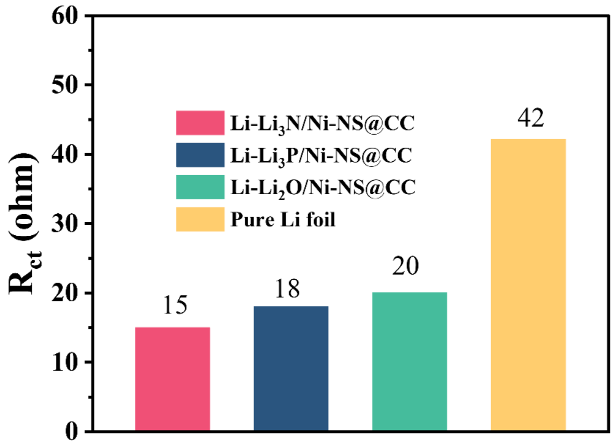

Fig. S26 The comparison of R_{ct} of the above electrodes after cycling

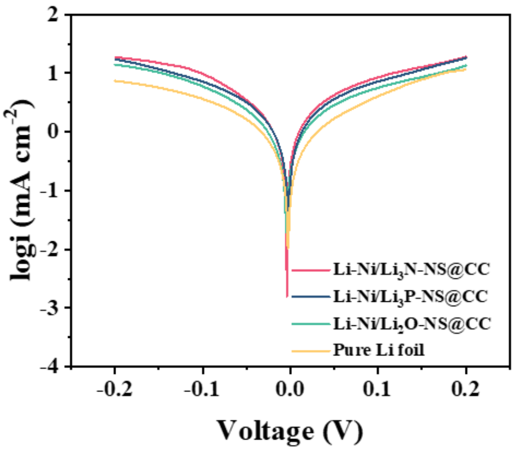

Fig. S27 The Tafel plots of symmetrical batteries with the Li-Ni/Li_2O-NS@CC, the Li-Ni/Li_3N-NS@CC, the Li-Ni/Li_3P-NS@CC, and pure Li electrode after 20 cycles

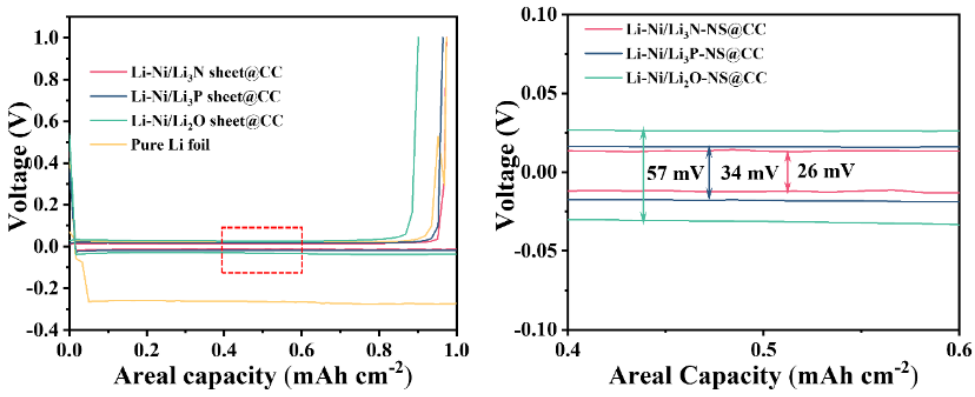

Fig. S28 a Voltage profiles of the Li-Ni/Li_2O-NS@CC, the Li-Ni/Li_3N-NS@CC, the Li-Ni/Li_3P-NS@CC, and pure Li electrodes during 2nd Li plating/stripping at 1 mA cm^{-2}, 1 mAh cm^{-2} and **b** the enlarged view

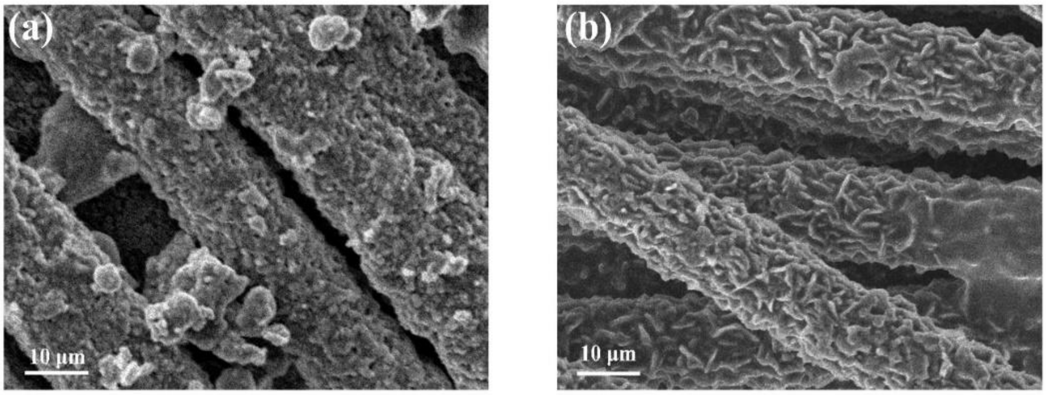

Fig. S29 SEM images of **a** the Li-Ni/Li_2O-NS@CC and **b** the Li-Ni/Li_3P-NS@CC electrode after stripping 20 mAh cm^{-2}

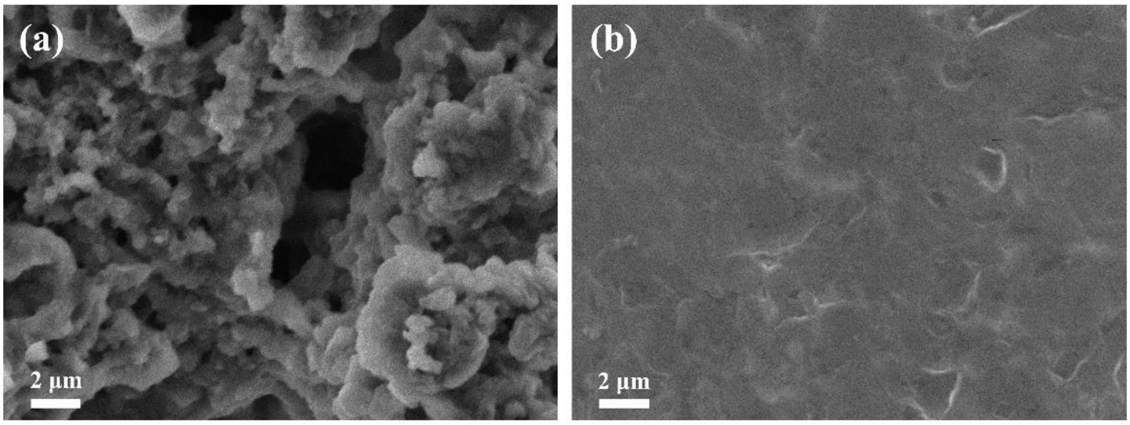

Fig. S30 SEM images of **a** pure Li foil and **b** Li-Ni/Li_3N-NS@CC electrode after 40 cycles in full batteries

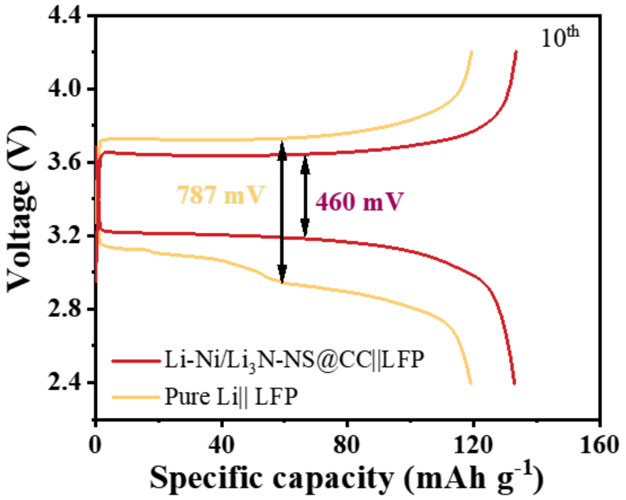

Fig. S31 Galvanostatic charge-discharge profiles of Li-Ni/Li_3N-NS@CC||LFP and pure Li||LFP full batteries at 2 C

Table S1 The density and porosity of each electrode

Samples	Density (g cm^{-3})	Porosity (%)
NiO-NS@CC	0.492	73.4
Ni_3N-NS@CC	0.537	75.1
Ni_5P_4-NS@CC	0.736	70.6
